# Cyclic Stress at mHz Frequencies Aligns Fibroblasts in Direction of Zero Strain

**DOI:** 10.1371/journal.pone.0028963

**Published:** 2011-12-16

**Authors:** Uta Faust, Nico Hampe, Wolfgang Rubner, Norbert Kirchgeßner, Sam Safran, Bernd Hoffmann, Rudolf Merkel

**Affiliations:** 1 Institute of Complex Systems, ICS-7, Biomechanics, Forschungszentrum Jülich GmbH, Jülich, Germany; 2 Department of Materials and Interfaces, Weizmann Institute of Science, Rehovot, Israel; Massachusetts Institute of Technology, United States of America

## Abstract

Recognition of external mechanical signals is vital for mammalian cells. Cyclic stretch, e.g. around blood vessels, is one such signal that induces cell reorientation from parallel to almost perpendicular to the direction of stretch. Here, we present quantitative analyses of both, cell and cytoskeletal reorientation of umbilical cord fibroblasts. Cyclic strain of preset amplitudes was applied at mHz frequencies. Elastomeric chambers were specifically designed and characterized to distinguish between zero strain and minimal stress directions and to allow accurate theoretical modeling. Reorientation was only induced when the applied stretch exceeded a specific amplitude, suggesting a non-linear response. However, on very soft substrates no mechanoresponse occurs even for high strain. For all stretch amplitudes, the angular distributions of reoriented cells are in very good agreement with a theory modeling stretched cells as active force dipoles. Cyclic stretch increases the number of stress fibers and the coupling to adhesions. We show that changes in cell shape follow cytoskeletal reorientation with a significant temporal delay. Our data identify the importance of environmental stiffness for cell reorientation, here in direction of zero strain. These in vitro experiments on cultured cells argue for the necessity of rather stiff environmental conditions to induce cellular reorientation in mammalian tissues.

## Introduction

Cell adhesion and the corresponding mechanical coupling to the environment are prerequisites for the survival and functionality of numerous cell types. For animal cells the central structures for adhesion are focal adhesion sites comprising numerous proteins. These complex dynamic structures couple the extracellular matrix (ECM) proteins to intracellular actin bundles, mostly so called stress fibers [1], [2]. Thereby, focal adhesion sites mediate not only strong adhesion to the substrate, but are also able to bi-directionally transmit signals between the ECM and the cytoplasm [1], [3].

The direct interaction between the cell and the ECM enables cells to actively sense and to respond to varying environmental conditions and thereby to maintain mechanical homeostasis [4]. This is a fundamental feature, since adherent cells not only exert active cellular forces on the ECM but are also constantly exposed to various externally generated mechanical forces such as shear flow, uniaxial stress or strain. The last two mechanical forces are in the focus of this work. By remodeling their actin bundles and adjusting traction forces, cells continuously adapt to their dynamic mechanical environment. However, despite the importance of mechanotransduction for many physiological processes such as cell differentiation, proliferation or wound healing [5], [6], many aspects of the underlying mechanisms are still not understood.

When cells are exposed to strain resulting from environmental stretch, their actin bundles reorient and thereby change the transmission of stress through the filaments to maintain optimum mechanical conditions [7]–[11]. The orientational response of the cells differentiates between constant static and cyclically varying strain application. While it has been observed that under conditions of static or quasi-static strain certain cells orient parallel to the stretch direction [12], [13], the opposite occurs for cyclic strain at amplitudes of 8 to 20% and frequencies in a range of 0.25 to 1 Hz. Here, cells respond with a reorganization of actin bundles and cell shape alignment almost perpendicular to the direction of stretch [11], [14], [15]. These *in vitro* data agree well to *in vivo* orientations of vascular endothelial cells parallel to the direction of blood flow and therefore perpendicular to stretch direction ([16], [17].

To explain mechanically induced cell reorientation, i.e., cell reorientation under varying external stresses, De and Safran [8] developed a coarse-grained theoretical model. This model considers forces induced both actively by cells due to cytoskeleton rearrangement as well as passively by substrate stretch. Focusing on cells with bipolar morphologies, the model idealizes stationary adhering cells as force dipoles composed of two oppositely directed forces exerted at the cell extremities and their separation distance [18]. These force dipoles are able to change both their magnitude and direction, i.e. cell contractile activity and cell orientation, respectively. A major open question in this respect is if stress (force per area) or strain (material deformation) is the relevant control parameter. Since the experimental answer [19] was not known at that time, the model was formulated in both variants [8]. To explain the different responses to static or quasi-static and dynamic strain the model takes into account the fact that cells require a certain time to reorganize their actin fibers and focal adhesions. If the external strain varies slower than this reorganization time scale, cells reorient and reorganize the cytoskeleton to re-establish the optimal strain. This can result in a more parallel orientation to strain direction. At high frequencies, however, when the cyclic strain varies faster than the cells can reorganize, the cells cannot follow. The time-averaged reaction of the cell (appropriate at high frequency) leads to an orientation approximately perpendicular to the direction of strain. The theory assumes that cells minimize the forces acting on them by aligning at an angle where the time-varying strain is as low as possible; this enables them to obtain their optimal strain set point [8]. A molecularly based explanation is given by Hsu et al. [20]. However, since this effect competes with the elastic interaction of the force dipole with the stretched matrix that tends to align the cell in the parallel direction, the applied stresses must exceed a certain threshold in order to induce cell response.

Moreover, for a given cell, other processes can modify the mechanical response. Examples are the detailed architecture of the cytoskeleton, the metabolic state of the cell and unrelated chemical signals triggering cell activity. To describe cell behavior at a coarse-grained level, all these effects are lumped into an “effective temperature” that tends to randomize cell orientation [8]. Depending on the balance between randomization and cell matrix interaction, the threshold mentioned above can be more or less abrupt.

Many studies have been performed on the influence of different parameters such as cyclic stretch frequency or amplitude on cell orientation and the dynamics of this mechanoresponse. It is well accepted that the reaction of cells to cyclic stress is an “escape mechanism” to avoid stretch [21], [22]. The frequency of cyclic stretch has been shown to influence the alignment of vascular smooth muscle cells [23] and plays a distinct role in the reorientation time of fibroblasts [22]. Furthermore, the degree of reorientation perpendicular to the strain direction seems to correlate with the stretch amplitude [11], [24], with a threshold of approx. 3% strain [25], [26].

Here, we present quantitative experiments on large cell populations that answer the question of whether cells reorient at mHz frequencies in direction of low strain or low stress. mHz frequencies were applied to slow down cellular reorientation dynamics and because many cell types in mammalian systems are primarily affected by low frequencies [27], [28]. In our case, fibroblasts were isolated from soft umbilical cord stroma. In vivo these cells are most likely primarily affected by tension and deformation caused by embryonic movement of low frequency.

Stretching experiments with chambers specifically designed to define and to vary directions of zero strain upon cyclic stretch allowed the automated evaluation of hundreds of cells per experiment. Cellular response was evaluated at the level of reorientation of cytoskeletal stress fibers as well as of the entire fibroblast cell. Those evaluations revealed a clear reorientation in the direction of zero strain. Interestingly, the orientation of the overall cell shape follows the cytoskeletal reorientation with a temporal delay. At all analyzed stretch amplitudes, the distribution of actin stress-fiber orientations in our cell population are well described by the theoretical model [8]. Furthermore, the degree of reorganization was dependent on substrate stiffness, with softer substrates resulting in less reorientation and no cellular response on very soft ones.

## Results

### Analysis of chamber deformations upon uniaxial stretching

**Figure 1:**
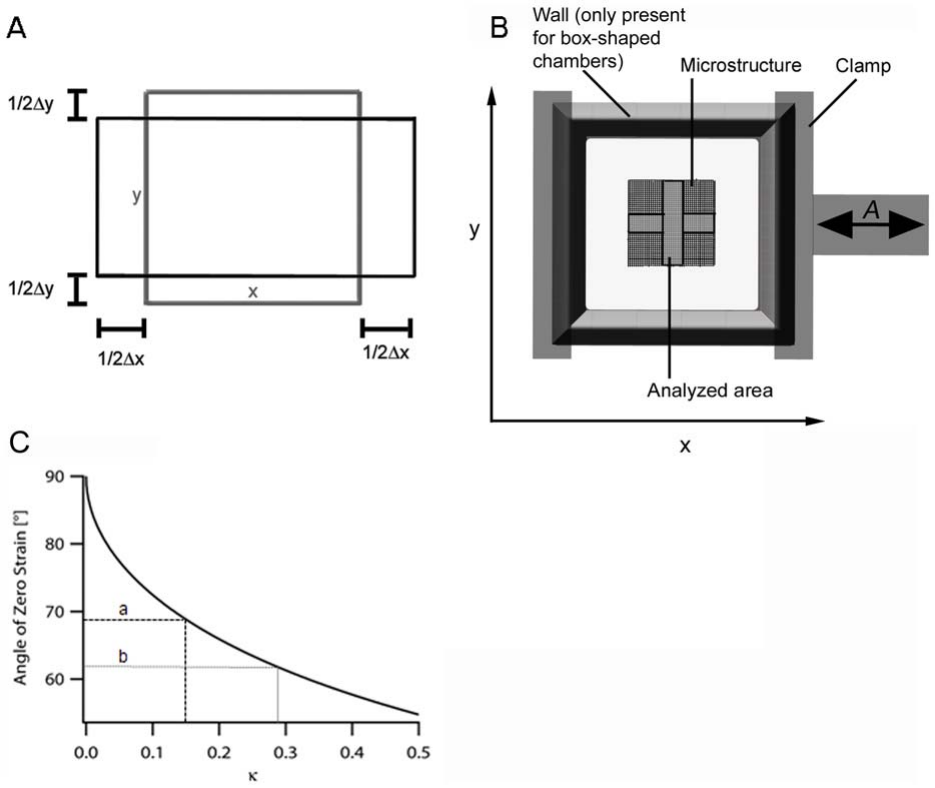
Interplay of elongation and contraction in stretching chambers. (A) An incompressible elastic material in uniaxial stress (i.e. forces act exclusively along one direction) exhibits a transversal shrinkage of exactly half the elongation along the direction of force. That is, upon elongation of an elastomeric film in x-direction, a film characterized by a Poisson's ratio of 0.5 is shortened in y-direction with Δy = ½ Δx. For adhered cells this results in tensile strain in x- and contractile strain in y-direction. (B) Scheme of the stretching design. An elastomeric chamber was formed of silicone rubber exhibiting a bulky 5 mm thick wall all around an approximately 400 µm thin film (Young's modulus 50 kPa, Poisson's ratio = 0.5). The chamber (length 20 mm, width 20 mm) was clamped on two opposite sides and stretched with controlled amplitudes A in x-direction. Exact substrate deformations applied to the cells were quantified by mapping a regular micro-pattern embedded in the silicone rubber film. All cells adhered in the indicated cross were analyzed. Ribbon-like chambers display an identical design except that bulky walls in x-direction (direction of stretch) are missing. (C) Dependence of zero strain direction on the measured transversal shrinkage factor κ (κ = −Δy/Δx). In theory κ can assume values from 0 (fully blocked perpendicular shrinkage upon film elongation) to 0.5 (free shrinkage upon elongation).

In our experiments elastomeric cell culture chambers were stretched along their long axes (Fig. 1B). The response of cells cultivated on the thin bottom (0.4 mm thickness) of such a chamber is dominated by its deformations. For clarification of the observed deformations we first recapitulate some basic results from classical continuum mechanics [29], [30]. In this field it is most important to distinguish between strain (deformations), which can be reliably measured in light microscopy, and stress (force per unit area).

The most basic description of material deformation (in the following, all deformations are assumed to be small) is the displacement vector field 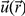 defined by the vectors that join the initial to the final positions of the material particles. In the following we will implicitly assume small strains. At larger strains more complicated definitions including non-linear terms should be used [31]. However, it is only at the highest strain used in this study that such nonlinear effects become notable, see discussion. Formally the elements of the strain tensor are given by
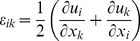
(1)where i and k symbolize any of the three spatial dimensions x, y, and z. Like any symmetric tensor the strain tensor can be diagonalized. Its eigenvectors define the principal axes of deformation and the corresponding eigenvalues give the relative length change along the respective principal axis. In uniaxial stress, i.e., in the case where forces act exclusively along one spatial direction, here arbitrarily the x-direction, a material is not only deformed in x direction by the relative amount of ε_xx_ but also in all perpendicular directions. For linear elastic and isotropic materials the perpendicular strain is given by Poisson's ratio ν
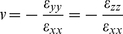
(2)Poisson's ratio is a material constant. For our elastomeric chambers we have found a value of 1/2, corresponding to an incompressible material (Fig. 1A) and a linear elastic response even for stretches of 50% [32]. Such a material behavior is indeed expected for a weakly cross-linked rubber [33].

A detailed description of the following chamber calibration can be found in the appendix (Appendix S1). Exploiting the microstructures molded into the chamber bottom (see Material and Methods) we could determine the displacement vector field 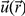 in the plane of the chamber bottom and thus quantify ε_xx_, ε_yy_ and ε_xy_ reliably. Here, we choose x to be the direction of external strain and z to be the surface normal of the bottom. Within the observation region of the chamber bottom the components of the strain tensor are assumed to be constant. Moreover, the deformation is almost shear free. To check this we analyzed the change of angle between the lattice vectors of the micropattern with increasing strain (up to 40%) on one chamber with and one without sidewalls. The strain anisotropies in both chambers were analyzed at five locations, one in the center of the micropattern and one each at the left, right, top, and bottom border of the pattern. We found on average an angular change of approximately 0.005° per % of strain. At 32% strain the angular change remained below 1.4°. Such comparably large changes were only reached at the upper and lower edges of the micropattern, at all other locations and at smaller strain, the changes were substantially lower. These and the following calibration experiments are explained in detail in the appendix (Appendix S1). In this case, the length change of a line within the surface of the bottom (i.e. with vanishing z-component) is given by
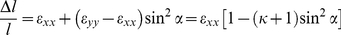
(3)where α denotes the acute angle formed by that line and the x-direction and 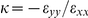. From the Poisson's ratio of the material one expects to find a value of 0.5 for κ. However, we experimentally obtained κ = 0.15 (with an uncertainty of 0.05, four chambers were calibrated independently). Elastomeric ribbon-like chambers lacking side walls in direction of stretch were also calibrated yielding κ = 0.29 (with an uncertainty of 0.05, two chambers were calibrated). Both types of chambers were used for subsequent experiments. Note that the experimentally observed transversal strains in the chamber bottom were therefore not given by Poisson's ratio. This apparent contradiction can be explained by transversal forces due to e.g. the side walls of the chamber. To quantify these, we must introduce the stress tensor.

The element 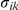 of the stress tensor is defined as the i-component of the force per unit area acting on an area element perpendicular to the k direction, where again i and k denote any of x, y, and z. For an isotropic elastic material with Poisson's ratio ν and Young's modulus E stress and strain tensor are connected by
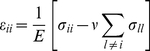
(4)and
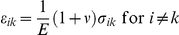
As there were no sizeable external forces in direction perpendicular to the chamber bottom (i.e. σ_zz_ = 0), we obtain by inverting Eq. 4
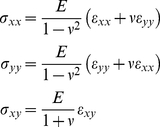
(5)This yields
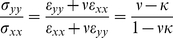
(6)For our case, this equation implies that tensile stresses act in both directions with a perpendicular stress (σ_yy_) amounting to 0.38 of the stress in the stretch direction for (ν = 1/2, κ = 0.15) and 0.25 for (ν = 1/2, κ = 0.29). These stresses are mainly caused by bending of the side walls of the cell culture chamber.

In contrast to stress which is tensile in all directions within the surface of the chamber bottom, strain can be tensile or contractile, i.e. lengths can be extended or reduced depending on direction. The direction of zero strain, α_0_, is given by the root of Eq. 3
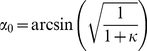
(7)Lines enclosing larger acute angles with the direction of external strain (x) are shortened, whereas all others are lengthened. This knowledge allowed us to deliberately tune α_0_ by modifying the wall thickness of our elastomeric chambers. For our box-shaped setup with κ = 0.15 we find α_0_ = 69°, whereas α_0_ for ribbon-like chambers was 62° (Fig. 1C).

### Cells as well as the actin cytoskeleton reorient in response to cyclic stretch

**Figure 2:**
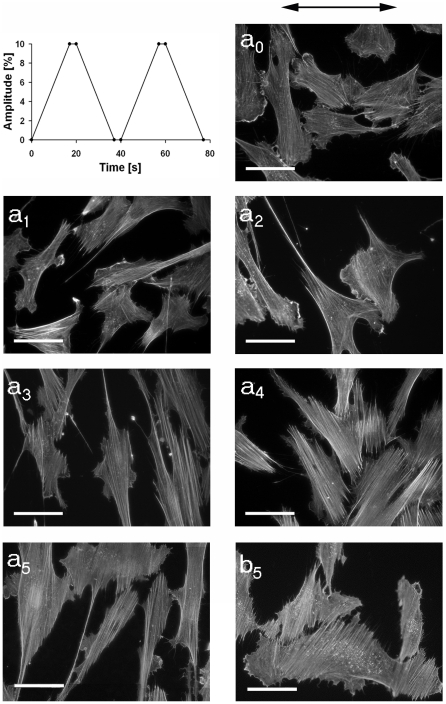
Cell reorientation upon cyclic stretch. Stretch cycles as indicated (top left) of various amplitudes (a_0_ (0%), a_1_ (4.9%), a_2_ (8.4%), a_3_ (11.8%), a_4_ (14.0%), a_5_ (32%), b_5_ (31.7%)) were applied to adhered cells for 16 hours. Subsequently, cells were fixed and stained for actin. The arrow indicates the direction of stretch. Note the induced cytoskeletal reorientation upon cyclic stretch. Frequencies used for each amplitude are given in table 1. Scale bars, 50 µm.

**Figure 3:**
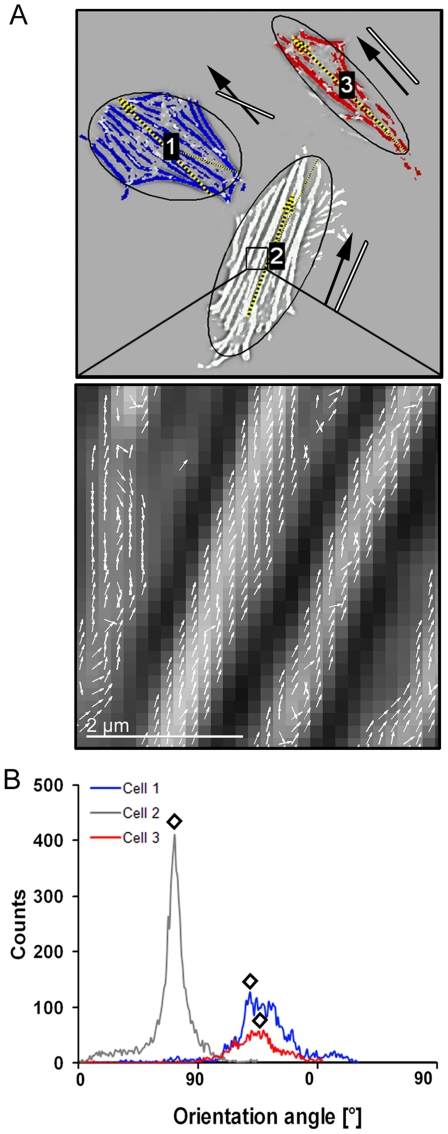
Determination of cell shape and cytoskeletal orientation. (A) Cells were stained for actin after stretch application and cell fixation. Every cell and its actin bundles were recognized by image processing. Actin bundle directions were determined at every single pixel as perpendicular direction to the grey value gradient (inlay). The major axis of the ellipse with the same normalized second central moments as the cell characterized the main cell orientation. Black arrows next to the cells indicate main actin orientation, and the white lines give the cell shape orientation. (B) Histogram of measured actin orientations of the cells 1 to 3 given in A. All values of each cell were used to determine the main actin orientation angle indicated for the three cells (diamonds). See also Table 3 or Figure 4.

Box-shaped elastomeric chambers were used, and adhered cells were stretched cyclically. Depending on the amplitudes, here denoted as a_i_, the frequencies for one complete cycle ranged between 52 mHz for a_1_ (4.9%) and 9 mHz for a_5_ (32.0%). After 16 hours of cyclic stretch cells were fixed and immunofluorescently labeled for actin. Already starting at amplitude a_1_, cell shape orientation as well as cytoskeletal orientation realigned in distinct angles relative to the direction of stretch (Fig. 2). These data were in good agreement with former studies [11], [14], [24]. In order to characterize average orientations and the distribution of both the cell shape orientation as well as the angles of the actin stress fibers for each amplitude, overlapping images were taken. Images overlapped over the entire elastomeric substrate in one line perpendicular to and another parallel to the direction of the applied stretch. Each cell within these stripes was identified and evaluated by image processing. Hereby we determined the corresponding ellipse of the cell shape, the orientation of its major axis and the orientation of actin bundles (Fig. 3A and 3B). High stretch amplitudes (amplitude a_4_) resulted in changes of the average aspect ratio (ratio of length to width) from 3.2 (s.d. = 2.3, n = 109) under control conditions to significantly different values of 5.3 (s.d. = 4.2, n = 300 cells) (Table 1). At the same time, the average angular spread of the cell shape orientations (s.d.) dropped from 27° for unstretched cells to approx. 23° after a_4_ stretch (Table 2).

**Table 1:** Average cell aspect ratios.

	εxx [%]	frequency [mHz]	aspect ratio	s.d.	n
**a_0_**	0.0	0.0	3.2	2.3	109
**a_1_**	4.9	52	2.8	1.6	88
**a_2_**	8.4	34	3.2	1.8	87
**a_3_**	11.8	25	5.9*	6.9	81
**a_4_**	14.0	21	5.3*	4.2	300
**a_5_ boxshape**	32.0	9	3.8	5.3	197
**b_5_ ribbon**	31.7	9	2.2	1.0	278

Aspect ratio of the cells stretched with indicated strain amplitudes and stretch frequencies. Aspect ratios are given with their standard deviations (s.d.). Asterisk = significantly different to a_0_ (P≤0.05). n = number of analyzed cells.

**Table 2:** Mean cell orientations.

	εxx [%]	mean orientation [°]		s.d. [°]	n
**a_0_**	0.0	43		27	771
**a_1_**	4.9	49		26	862
**a_2_**	8.4	53		24	273
**a_3_**	11.8	61		22	261
**a_4_**	14.0	61		23	168
**a_5_ boxshape**	2.0	65		22	180
**b_5_ ribbon**	31.7	61		23	221

Mean cell orientation angles in response to uniaxial cyclic stress of indicated amplitudes (ε_xx_ [%]). The standard deviation (s.d.) gives the cell angle spread within a sample. n = number of analyzed cells.

### Actin bundles orient in direction of low tensile strain

**Figure 4:**
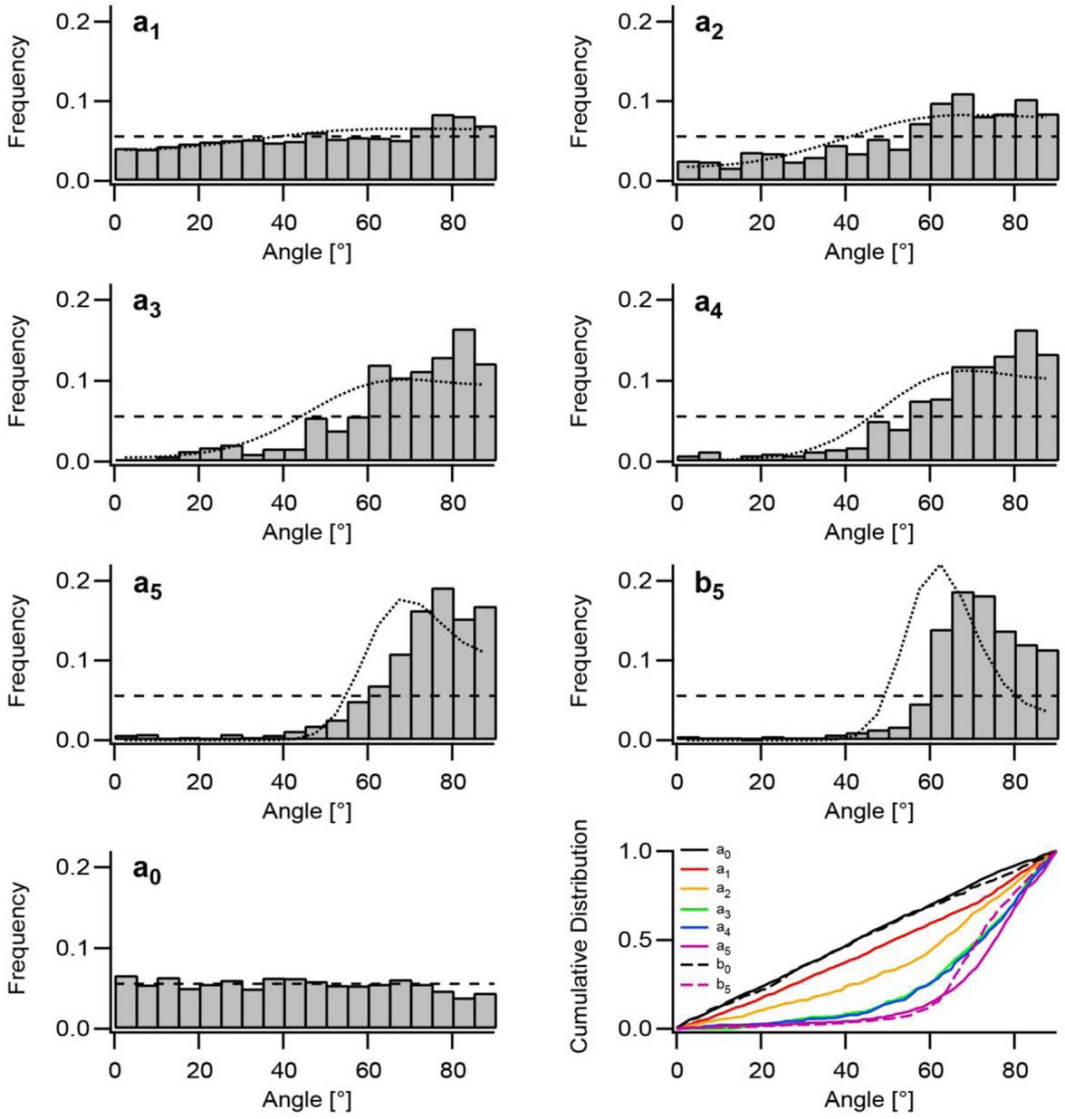
Orientation of stress fibers in response to uniaxial cyclic stretch. The predominant cytoskeletal orientations were determined for cells stretched for 16 hours at amplitudes a_1_ (4.9%, 1493 cells), a_2_ (8.4%, 662 cells), a_3_ (11.8%, 625 cells), a_4_ (14.0%, 397 cells), and a_5_ (32%, 772 cells) in box-shaped chambers (κ = 0.15) and at amplitude b_5_ (31.7%, 588 cells) on ribbon-like substrates (κ = 0.29). Control measurements in both types of chambers (a_0_ (0%, 1417 cells), b_0_ (0%, 640 cells, b_0_ only indicated as cumulative plot)) showed random distributions. Distributions are given as histograms and cumulative distributions (bottom right). The dashed line in each histogram indicates a random distribution. The dotted lines express the theoretical description of the measured actin angle distributions (see results section for details). All distributions differ significantly from each other according to Kolmogorov/Smirnov except a_0_ from a_1_ and a_3_ trom a_4_.

Since our analyses were based on large numbers of cells and a precise determination of both cell and stress fiber orientation, we were able to characterize both in more detail. For each cell, the peak actin bundle orientation angle (see Material and Methods section) was determined after 16 hours for a_0_ (ε_xx_ = 0%), a_1_ (ε_xx_ = 4.9%), a_2_ (ε_xx_ = 8.4%), a_3_ (ε_xx_ = 11.8%) and a_4_ (ε_xx_ = 14%) stretch amplitudes, respectively. All resulting peak actin orientation angles were plotted in 5° intervals (Fig. 4 and Table 3). While there was a homogeneous angle distribution for cells under unstretched conditions (a_0_), every amplitude from a_1_ to a_4_ significantly changed the actin angle distribution. In detail, already the relatively small substrate stretch of 4.9% reduced angle orientations in the 0 to 20° range relative to stretch direction by 27% (by 18% for 0–50°). At the same time angle orientations above the angle of zero strain (70° to 90°) elevated by 43%. At stretch amplitude of a_2_ cytoskeletal reorientations between 70° to 90° became increasingly prominent (+87% relative to random distribution). Correspondingly, angles in the range of 0–50° dropped by 45%. For a_3_ and a_4_ stretches, cytoskeletal orientations with an angle below 50° relative to the direction of stretch were rarely observed (reduction by 74% (a_3_) and 76% (a_4_), respectively). In parallel, actin orientations between 70 to 90° increased by 155% (a_3_) and 169% (a_4_). Since the total number of cells remained visibly unchanged during analysis, the effect was not due to induced cell death. Interestingly, we found that at the tested stretch amplitudes from a_1_ to a_4_, all cytoskeletal orientations from zero strain orientation (69°) to minimal stress direction (90°) were equally preferred.

**Table 3:** Distribution of actin angles.

	ε_xx_ [%]	mean orientation of peaks [°]	s.d. [°]	n
**a_0_**	0.0	44	26	1417
**a_1_**	4.9	50	26	1493
**a_2_**	8.4	58	24	662
**a_3_**	11.8	68	17	625
**a_4_**	14.0	69	18	397
**a_5_ boxshape**	32.0	73	15	772
**b_5_ ribbon**	31.7	68	19	588

Distribution of actin angles. Mean orientation of peaks denotes the mean of the peak angles of all analyzed cells and is given for the indicated stretch amplitudes (ε_xx_). Numbers are given with their standard deviation (s.d.). n = number of analyzed cells.

In order to also apply sizeable strains to cells oriented along the direction of minimal stress (i.e. 90°) we further increased the stretch amplitude to as much as 32% and applied this amplitude to our box-shaped chamber (a_5_) and also to our ribbon-like chambers characterized by a κ-value of 0.29 (Fig. 4, b_5_). For the ribbon-like chamber this resulted in a shift of the zero strain direction from 69° to 62° and in a contractile strain perpendicular to the stretch direction of as much as 9.2%. Actin angle distributions of cells stretched under these conditions in box-shaped chambers (a_5_) were reduced by 81% compared to random distributions for the angle range of 0 to 50° and increased by as much as 401% for angles above the angle of zero strain (70 to 90°). However, those angles were still almost equally, not significantly differently distributed. In contrast, actin distributions at b_5_ show a clearly peaked distribution with its maximum shifted towards direction of zero strain (Fig. 4). Cells with orientations of low stress and therefore high contractile strain are significantly reduced in number compared to a_5_ although stretch frequency and amplitude were unchanged. Here, angles in the range of 70° to 90° were reduced by 10% and even by 28% for the minimal stress range of 80° to 90°. Prolonged cyclic stretch experiments (24 h) for intermediate amplitudes as e.g. a_1_ revealed identical cytoskeletal angle distributions as found for 16 hours (data not shown). This shows that angles reached a steady state distribution already after 16 hours.

### Theoretical description of the measured distributions of actin angles

De and Safran [8], [10] have presented a generic model that takes into account forces that operate on the cell via an effective free energy experienced by a cell during one strain cycle. In their theory, a cell is modeled as an active force dipole that adjusts its contraction strength to maintain an intrinsic value of either stress or strain in the matrix adjacent to the cell. The effective energy experienced by a cell is defined as the work done on it with respect to the intrinsically “preferred” value of stress or strain. Reactions of cells are derived from this effective free energy by standard methods of statistical physics. As can be clearly seen at high strains (Fig. 4), the cells studied here do not orient towards minimum stress direction (90°), instead they preferred directions closer to the minimum strain direction (69° in box-shaped chambers and 62° on ribbons). Because of this observation, for the given stretch amplitudes we can exclude stress as set-point for cell force homeostasis and analyze our data using the equations that consider strain as set-point (Eq. F1 in [8] and Eq. 6 in [10]). More details of the model and its comparison with the data can be found in the discussion section. An in-depth quantitative discussion of the results of stress or strain as set-point for cellular reorientation can be found in [7].

The effective free energy is minimized with respect to the cell contraction which is equivalent to the zero force condition that balances the cell's reaction to external strain. Furthermore neglecting contributions of the matrix in which the cell is embedded, we arrive at the following equation for the effective free energy F (time averaged over one cycle of the external stretch).
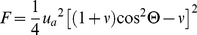
(8)Here u_a_ is proportional to the amplitude of the applied cyclic strain and is multiplied by a parameter of the theory that describes the cell's tendency to follow such orientation cues multiplied by the set point stress.

The theory assumes ideal material behavior under uniaxial stress (i.e. no transversal forces due to stiff sidewalls). To rectify this idealization of our experimental situation we used the measured perpendicular shrinkage ratio κ instead of the Poisson's ratio ν. Only this choice ensures that the effective free energy F is minimal in the direction of zero strain α_0_, cf. Eq. 7.

However, real cells do not perfectly align along this direction. Instead a substantial scatter is observed (Fig. 4). This scatter arises from noise in the system (see [10] and the discussion) which can be modeled as an effective temperature and a Boltzmann distribution function of angles. This yields the distributions of cytoskeletal angles, P(θ):
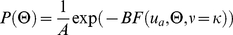
(9)where B is the inverse effective temperature. A is a trivial normalization constant. Note that the only unknown parameter in these distributions is B which we determine by a simultaneous fit of Eq. 9 to all measured distributions. This fit is done once and the same value is used for all amplitudes of strain. The resulting theoretical distributions are displayed as dotted lines in Fig. 4 and describe to a good extend the experimental data, cf. also the discussion section.

### Cell orientation follows cytoskeletal reorientation with a temporal delay

**Figure 5:**
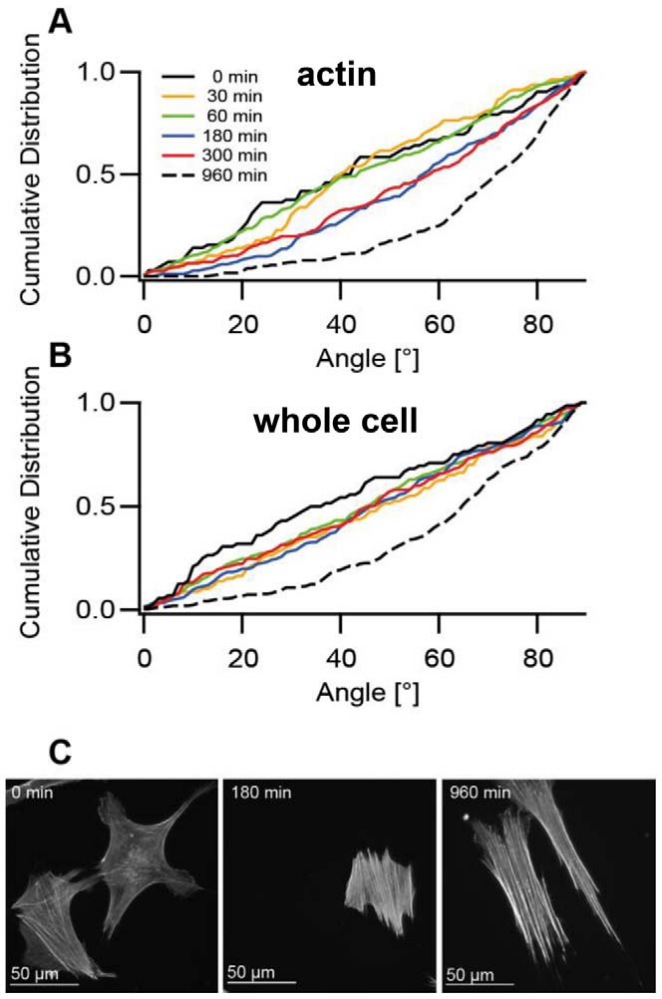
Time evolution of cytoskeletal (A, actin) and cell orientation (B, whole cell) upon cyclic strain of 11.8% amplitude (a_3_). Control data (0 min) were taken on cells cultivated for 5 hours without applied strain. The final reorientation distribution in A and B is indicated by the dashed black line. Cell numbers in order of increasing duration: 72, 80, 227, 187, 159, and 261 cells. Note that for (A) all time points from 180 min and higher differ significantly from control while in (B) all distributions from 30 to 300 min equal random distributions (p = 0.05 according to Kolmogorov-Smirnov test). The control distribution for 0 min is slightly shifted due to the relatively low number of cells evaluated for this time point. In (C) exemplary micrographs of cells stretched with amplitude a_3_ for indicated times and subsequently labeled for actin are shown. Staining was done in parallel and microscope settings were kept identical to allow comparison of labeling intensities.

To follow the reorientation behavior over time the average cell shape orientation angles were compared to the most probable (peak of the distribution) actin bundle orientations at a_3_ stretch amplitude as at various time points. While the mean angles for both revealed no preferred orientation within the first 60 minutes, a significant (p = 0.05) reorientation of the actin bundles was found after 3 hours (Fig. 5A). A further slow reorientation process finally led to the new steady state of reoriented actin bundles after 16 hours. A different type of behavior was found for the overall cell shape orientation angles (Fig. 5B) where no significant reorientation processes were observed after 3 and even after 5 hours of cyclic stretch. Only after several additional hours of stretch application, the cell shape orientations finally followed the actin reorientation. The faster reorientation of actin cytoskeleton compared to cell shape is illustrated in Figure 5C. The delay in cell shape orientation by several hours presents important evidence that cell shape follows the actin bundle orientation.

### Cytoskeletal reorientation is associated with enhanced actin bundle formation

**Figure 6:**
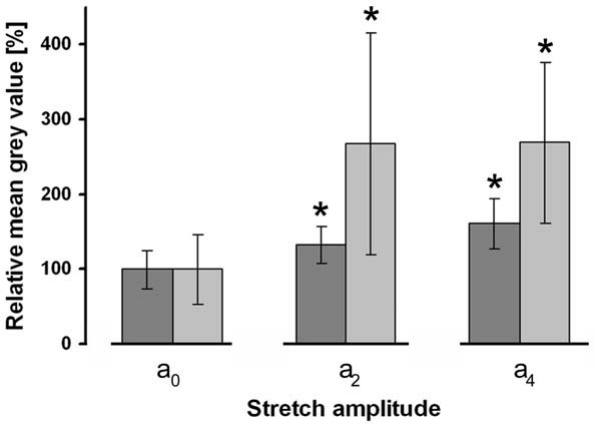
Actin and vinculin concentrations increase in stretched cells. Cells were cyclically stretched with amplitudes a_2_ (8.4%) and a_4_ (14%), respectively, and immunofluorescently labeled for actin and vinculin. Mean grey values for actin (dark grey bars) and vinculin (light grey bars) were determined and are given relative to the control (a_0_) values. For each stretching experiment actin bundles from 20 cells and 100 FAs from 5 to 7 cells were evaluated. Asterisk = significantly different to a_0_ (P≤0.05).

Quantitative immunostaining experiments for actin and vinculin were performed after 16 hours at various stretch amplitudes. Actin staining significantly increased by 40% at a cyclic stretch of amplitude a_2_. An increased stretch amplitude of a_4_ resulted in an additional increase by 20% (Fig. 6, see also Fig. 5C for a_3_). An even more obvious increase in intensity was observed for focal adhesions stained by vinculin. Here, cyclic stretch by a_2_ as well as a_4_ amplitude resulted in an increase of vinculin staining by more than 250% (Fig. 6). Interestingly, neither the size nor the average number of focal adhesions per cell changed significantly over time (not shown).

**Figure 7:**
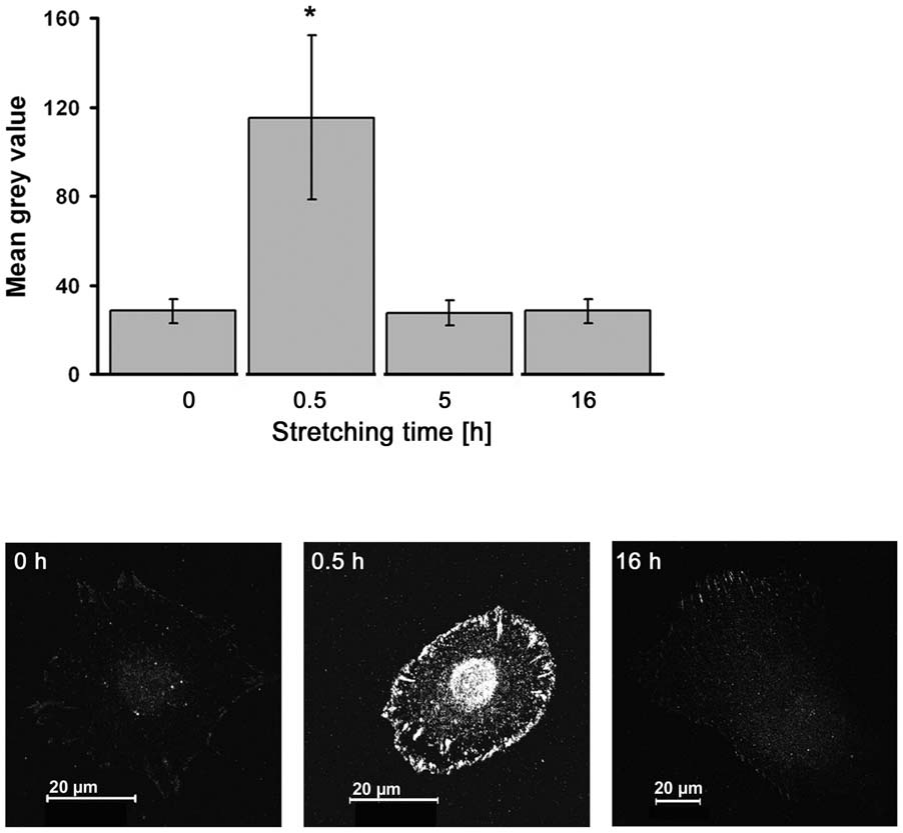
Tyrosine phosphorylation during stretch application. Cells were cyclically stretched with amplitude a_3_ (11.8%) and immunofluorescently stained for phospho-tyrosine after indicated durations. All time points were stained simultaneously. Mean grey values are given. n = 50 focal adhesions from 5 to 6 different cells. Asterisk = significantly different to initial values (0 h) (P≤0.05). Typical images analyzed for phospho-tyrosine staining of FAs are indicated below the graph. Here, microscope settings were kept identical for all images and show therefore basically no phosphorylation at time points 0 h and 16 h.

Reorientation can be regulated by and go along with various processes as protein phosphorylation, protein degradation or elevated expression. Since dynamic processes affecting the actin cytoskeleton and especially focal adhesions can be heavily regulated at the level of tyrosine phosphorylation, we additionally tested the time course of total tyrosine phosphorylation levels within focal adhesions upon cyclic stretch. While unstretched cells exhibited low levels of phospho-tyrosine, phosphorylation increased drastically by a factor of four within the first 30 minutes of applied cyclic stretch (Fig. 7). Within the next 90 minutes (not shown) cells eventually returned back to a homeostatic state of tyrosine phosphorylation and stayed constant at this original level for all subsequent time points analyzed. Total phospho-tyrosine levels therefore peaked clearly before actin reorientation was detectable.

### The cellular mechanoresponse depends on substrate stiffness

**Figure 8:**
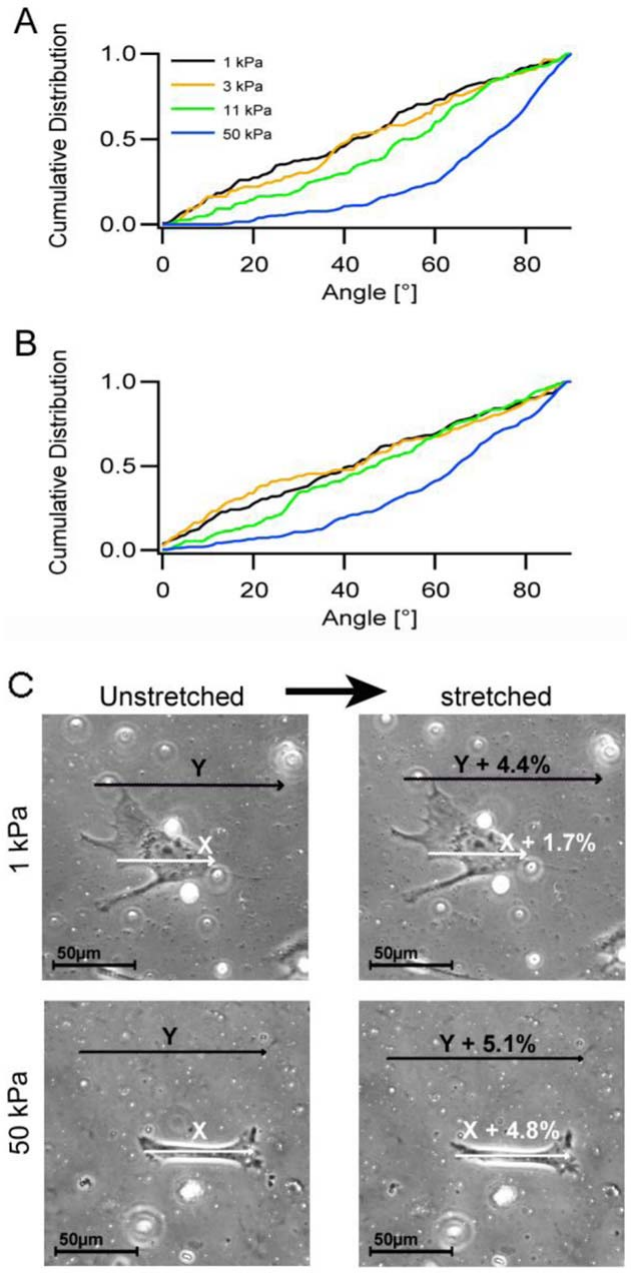
Effect of substrate elasticity on mechanoresponse induction. Cells on stretch chambers of ∼1 kPa, 3 kPa, 11 kPa and 50 kPa elasticity were stretched for 16 hours with amplitude a_3_ (11.8%). Subsequently, cells were fixed, stained for actin and main actin orientation angles (A) as well as main cell orientations (B) were determined. n equals 145, 86, 152 and 261 cells for Young's moduli of ∼1 kPa, 3 kPa, 11 kPa and 50 kPa, respectively. (C) Single cell analysis on cells grown on ∼1 kPa and 50 kPa substrates, respectively, before and after the indicated substrate stretch (Y, black arrow). Substrate elongations under the cell (X, white arrow) were determined. The indicated percentage is the effective substrate elongation. Note the strongly reduced substrate stretch under the cell on ∼1 kPa substrates. n = 2 cells on ∼1 kPa substrate and 3 cells on 50 kPa substrate.

Our detailed analysis of cytoskeletal reorientations argued for a relative insensitivity of cells to low tensile strain and thus also low forces. In order to analyze this hypothesis in more detail, cells were incubated on various substrate elasticities and subsequently cyclically stretched for 16 hours at identical amplitudes (a_3_, 11.8%) and frequencies (25 mHz). Softening the substrate from the 50 kPa used in the experiments described above down to 11 kPa, and further to elasticities in the range of 3 kPa and approximately 1 kPa, respectively, resulted in reduced forces applied to every single cell at constant substrate deformation. Cells adhered to the two softest substrates, respectively, were entirely unaffected by cyclic stretch. They displayed a mean orientation angle of around 45° (Fig. 8A). At substrate elasticities of 11 kPa the first significant reorientation processes of actin bundles were observed with similar mean orientation angles as found for a_1_ to a_2_ stretch amplitudes on the 50 kPa substrates described above. Interestingly, and as seen before for the time evolution of the reorientation processes (Fig. 5), the cell shape orientation was affected very little and followed actin reorientation only on stiffer substrates (Fig. 8B).

### The cellular mechanoresponse shows an apparent force threshold

Furthermore, precise measurement of the cell elongations of cells adhered to ∼1 kPa and 50 kPa substrates were determined upon a_1_ stretch. While the substrate strain was completely unaffected by its elasticity (Fig. 8C and Table 4), simultaneous elongation of adhered cells was mainly detected on stiff substrates. On very soft substrates cell lengths changed only slightly (approximately by 35% of applied substrate stretch) while cells stretched on stiffer 50 kPa substrates followed substrate strain on average by more than 90%. Since only minor cell elongation and no reorientation was found on ∼1 kPa soft substrates upon a_1_ substrate elongation, forces applied by the cell to the substrate in order to withstand the substrate stretch were approximated based on the assumption that substrate stretch under an undeformed cell is mechanically equivalent to shrinkage (i.e. contraction) of a cell on an undeformed substrate as described in the supplementary material (Material S1). Cell force estimations for seven independent cells resulted in force values in the range of 0.2 nN (s.d. 0.06 nN) for every focal adhesion and a sum of absolute values of all forces of 15 nN (s.d. 7.8 nN) (see Material S1). In the case of cells cultivated on ∼1 kPa substrates these forces amounted to more than 0.2 nN for single focal adhesions or 15 nN for the entire cell. On 50 kPa substrates, the force necessary to counteract the a_1_ substrate elongation would be in the range of approx. 9 nN (s.d. 3 nN) for every focal adhesion and a sum of absolute values of all forces of 1000 nN (s.d. 700 nN)(n = 14 independent cells). In this case, it is obvious that the cell can't counteract these substantial forces and is strained as a result. This cellular stretch in turn is the most likely cause for the induction of mechanoresponse. Please note that this force threshold very likely depends on substrate stiffness.

**Table 4:** Influence of substrate stiffness on cell property during stretch.

E [kPa]	εxx [%]	cell elongation [%]	n
∼1	4.0	1.6	2
50	4.6	4.3	3

Cell elongation during indicated stretch amplitude (ε_xx_ [%]) on substrates with different stiffnesses, E. n = number of analyzed cells.

## Discussion

Several recent studies have analyzed the effect of external mechanical forces such as shear stress and stretch on cells [11], [24], [34]. In these studies it was shown that cells exposed to cyclic stretch responded by reorganizing the actin cytoskeleton and by aligning the cell shape perpendicular to the direction of stretch [11], [14], [15]. As already assumed in [15], the orientation of cells in response to uniaxial stretch is determined by the substrate strain. The direction of zero strain, in which cells undergo no deformation, is strongly dependent on the material properties and on the characteristics of the stretching device. Therefore our experiments were performed using a carefully characterized micro-patterned stretching chamber with well-defined deformations (Appendix S1). The chamber design resulted in a zero strain angle of 69° relative to the direction of stretch and could be changed to 62° by modifying the chamber into a ribbon-like stretching device. This knowledge is indispensable for the interpretation of the reorientation data obtained in stretching experiments.

Most previous studies were based on relatively low numbers of cells and typically do not distinguish between cell shape orientation and cytoskeleton orientation [15], [22], [35], [36]. An additional strong advantage of the present study is therefore the automated evaluation of several hundreds of cells for each set conditions, allowing quantitative analyses with high statistical significance. Moreover, we used image processing methods to measure both, the predominant orientation of the cytoskeleton by determining the direction of actin bundles, as well as the orientation of the overall cell shape. These tools enabled a quantitative analysis of the reorientation process that is statistically meaningful.

In our study we applied various amplitudes at intermediate frequencies of 9 to 52 mHz. Most other studies have either applied frequencies in the order of 0.2 to 1 Hz or quasi-static strain. Our actin and cell shape angle distributions clearly argue for a similar cellular behavior at low mHz and fast 1 Hz frequencies. However, mHz frequencies might be the reason why reorientation processes for umbilical cord fibroblasts used here, reached a stable equilibrium only after 16 h compared to 1 to 6 h described for higher frequencies. The literature additionally provides evidence that reorientation rates are cell type specific [22], [24], [26]. Comparison of different systems is therefore difficult. Additionally, the density of cells most likely influences not only the orientation rate but also the entire mechanoresponse, since cell-cell contacts provide further pathways for mechanotransduction. Cadherins for example are able to directly transmit forces to the cytoskeleton [37]–[39]. In this work, the cell density was kept rather low to analyze reorientation processes of mainly separated cells.The prolonged time span till the new steady state was reached additionally allowed us to explicitly characterize the time course of actin as well as whole cell reorientation processes.

Although cells reoriented both in experiments at mHz frequencies performed here and high frequency analyses of other groups [11], [14], [15] it remains an open question whether reorientation into zero strain direction is a frequency independent process. The exact characterization performed here depended on the development of two different chamber designs both accurately characterized for κ. This enabled us to tune the zero strain direction from 69° to 62° with a resulting peaked actin angle distribution in this direction of reoriented cells. Experiments described in Refs. [11], [14], [15] argue for an also frequency dependent reorientation but whether cells reorient also in zero strain direction at high frequencies or prefer a minimal stress direction remains to be shown and is certainly out of the scope of this manuscript. Furthermore it needs to be mentioned that we decided to keep the stretch velocity stable for all amplitudes to exclude this parameter from putative explanations for observed cell behavior. However, amplitude and frequency are therefore not fully uncoupled in our experiments making it difficult to draw strong conclusions for that specific aspect of cell reorientation.

In good agreement with other studies [11], [24] we find that the extent of alignment presented in the results section correlated with the stretch amplitude but did not equally affect all cells. For example, at 4.9% external strain, cells oriented between 25° to 65° to the stretch direction before experimental start did not reorient, although the zero strain angle was 69°. Upon increasing the stretch amplitudes, this angular range decreased and moved closer to the zero strain angle. Interestingly, the effective substrate elongation applied to the cells adhered in such angles relative to the applied stretch was always in the range of 0 to approximately 3.5%. This behavior could be explained by the hypothesis that these cells may tolerate a certain elongation without inducing a mechanoresponse. This raises the question whether a threshold for the induction of a mechanosresponse exists, which can either be a force or a stretch, i.e. elongation of the cell. A stretch amplitude threshold has already been proposed for smooth muscle cells [26] bovine aorta endothelial cells [25], fibroblasts and osteoblasts [24]. These researchers found no cell realignment when the applied axial stretch (under the cell) was below 3%.

Several studies including the present, quantitative measurements of cell orientation distributions, have reported the existence of a threshold value of the applied strain, above which, the cell orients nearly perpendicularly to the strain direction (or as in the present case, in the zero-strain direction) ([22], [24], [26]. Some studies have found that at lower stress values the cells remain randomly oriented, although experiments in three-dimensional collagen matrices have reported parallel orientation for very slowly varying stresses [40]. As presented in more detail in Refs. [10], [41], the threshold may be due to a competition of the linear and non-linear response of the cell to the applied stress. The linear response arises from the interaction of the force dipole with the applied stress (analogous to the interaction of an electrical dipole with an electric field). This tends to align the dipole parallel to the field and gives a contribution to the average cell angle that is linear in the stress.

However, cell behavior always contains a significant degree of randomness. This stochastic contribution has very diverse causes as, e.g., the probabilistic nature of the physicochemical reactions during mechanosensing or inhomogeneities in the cell population. For the sake of simplicity all these effects are treated here as noise or an effective temperature. In a wide range of parameter values this noise term overwhelms the parallel orientation [10].

There are also terms in the response that arise from cell activity; in the limit of high frequencies, these contribute a response of the cell angle that is quadratic in the applied stress and tends to align the cell perpendicular to the field (for cells that are governed by stress) or in the zero strain direction (for cells governed by strain). Thus, for small applied stress, the linear terms will dominate and the cells will orient parallel to the applied stress or randomly if the effective temperature (or noise) is large. For larger values of applied stress above a threshold value, the quadratic term will dominate and the cell (if the noise is not too large) will orient either perpendicular to the applied stress (for cells governed by stress) or in the zero strain direction (for cells governed by strain). In this model, the threshold value of the stress is proportional to the ratio of the linear to quadratic responses.

Comparing measured data and theory one realizes that the maxima of the measured distributions do not exactly coincide with the angles of zero strain but are shifted to higher angles by about 10°. The reduced Χ^2^ value of the fit of 11.1 is to 73% due to this peak shift. Apart from that shift, the results of the fit and the measured data coincide quite well. Even the shift of the peak induced by changing the chamber type is captured correctly.

Obviously, the assumptions of the theory are to some extents an over-simplification of the experimental situation. First, in the experiment cells are cultivated on strained substrates whereas in the theory they are assumed to be embedded in a strained matrix. Second, the cells experience stresses in x and y direction (cf. Eq. 6) whereas the theory assumes purely uniaxial stresses. Third, the theory has been derived for small strain whereas the experiments have been performed at finite strain of up to 32%. Finally, fourth, in our derivation of Eqs. 8 and 9 we neglected cell matrix interactions to keep the numbers of fit parameters low.

Aspects one and two might easily alter the exact distribution of cytoskeletal angles but it is highly unlikely that going from bulk to the surface or adding strain in a second direction could cause a significant shift of the peak away from the direction of zero strain because even under these modified conditions the zero strain direction is the only orientation with distinct and special properties. As exactly such a shift of the peak is the most obvious deviation between data and theory, we believe that these first two effects are of minor importance. The third aspect, finite strain, indeed causes a rotation of the angle of zero strain towards higher values with increasing strain values. To first order in strain, the amount of rotation is given by 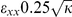. This expression amounts to at most 2.5° for our experiments. Thus finite strain effects may be contributing but are alone not sufficient to explain the angular shift of about 10°. An analysis of the free energy functional given as equation 6 in [10] shows that cell matrix interaction can indeed induce shifts of this size towards higher orientation angles. At high dimensionless amplitudes and high repetition frequencies a dimensionless matrix interaction parameter of 0.24 would be sufficient to explain the observed peak shift. If the cell is to some extent able to follow the oscillating strain (i.e., finite instead of infinite dimensionless frequency) even lower values of the matrix interaction parameter are sufficient.

Taken together, within the framework of the theory of Safran and De [10] the peak shift of about 10° towards higher angles can be explained by cell matrix interaction with perhaps some small contribution due to finite strain. However, it should be kept in mind that this analysis is based on data collected within a certain range of parameters. In our opinion, variation of the frequency in a large interval is needed in future to further test the limits of the model of Safran and De.

Moreover, from Eq. 6 in [10] it follows that, if cells were controlled by stress, only very specific, and thus unlikely, combinations of the parameters of the theory could result in angular distributions exhibiting a peak at intermediate angles. In this improbable case peaked distributions depend entirely on matrix interactions. Their position depends strongly on amplitude. In detail, at constant frequency and identical substrate we expect the peak position to satisfy
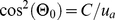
(10)where θ_0_ is the angular position of the maximum, u_a_ the amplitude applied and C a positive prefactor depending on frequency and matrix interaction. Thus, in the case of stress control the position of the peak depends strongly on amplitude, whereas in the case of strain control it should be almost independent of amplitude. Our results on cells in box-shaped chambers don't show altered preferential directions at different amplitudes (cf. Fig. 4). However, the observed width of the maxima and their proximity to 90° makes this comparison difficult. Therefore, in preliminary experiments we strained cells on a ribbon shaped chamber with lower amplitude (14% instead of 32%) while keeping the repetition frequency constant at 9 mHz. Assuming the unlikely case of stress control one would expect a shift of the maximum from the observed 70° to 60°. The distribution of cytoskeletal angles observed at 14% and 9 mHz is very broad, comparable to the results for a_2_ in Fig. 4 (data not shown). Thus no maximum at such comparatively low angles was seen. Therefore, we conclude that these cells at mHz frequencies reorient under strain control. That is, they sense substrate deformations.

Our reorientation analyses for cells grown on various elasticities clearly show a very prominent influence of substrate rigidity on cell reorientation response with a remarkable weakening of the response on softer substrates and a correspondingly higher threshold strain for the initiation of mechanoresponse. In this context it is instructive to calculate the mechanical forces experienced by individual focal adhesions and the entire cell at this threshold. On very soft, approximately 1 kPa substrates, cells were negligibly elongated upon 4% stretch amplitude. Consequently, they did not respond to stretch by reorienting cell shape or actin bundle orientation. Therefore, forces acting on cells under these conditions were below the threshold necessary to induce mechanoresponse. In contrast, on 50 kPa substrates, the applied chamber stretch of approximately 5% and the resulting cell elongation were almost identical. These cells reoriented in response to stretch, signifying that the forces in this case were above the threshold tolerated by the cells. Our estimates indicate a force range between 15 nN (estimations for the ∼1 kPa substrates, where no reorientation occurs) and 1000 nN (estimations for the 50 kPa substrates with complete cell reorientation). However, the lower bound is presumably much higher, as we found no reorientation for cells undergoing an 11.8% stretch (a_3_) on 3 kPa substrates. The same was found, as given above, on cells experiencing a 3% stretch on 50 kPa substrates. In summary, on very soft substrates only minute forces are applied to cells which are insufficient to stretch the cell by approximately 3%. On higher stiffnesses, the cellular contractile apparatus is much better developed. However, at the same time much higher forces can be applied by identical substrate strains eventually resulting in cell stretching. Such a stretch subsequently induces cellular reorientation in direction of zero strain.

The theory that is used here to compare with the experiments measuring the orientational distribution assumes that cell mechanosensitivity is well established; in that case, the cell responds both to internal regulatory cues that favor contractility, described by a force dipole moment P*, as well as to the appropriate component of the stress in the substrate right outside the cell as sensed by the focal adhesions [7], [8], [42]. However, on soft substrates, the mechanosensitive apparatus of the cell is not well established; focal adhesions are replaced by small, sometimes unstable, focal contacts and the stress fibers may be significantly less contractile and fewer in number. In that case, the model for the stress fiber orientational distribution is not valid. The sometimes severe reduction of cell contractility and polarization on soft substrates may occur because the alternative – of well-established contractile stress fibers and focal adhesions – would merely dissipate the cell energy into deforming the relatively soft substrate, with no functional advantage [8], . A simple physical model of this effect considers the forces due to both cell activity and to substrate elasticity; these linear restoring forces can be derived from the gradient of an effective energy, *F*, that can be written as
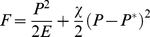
(11)where *E* is related to the Young's modulus of the substrate and χ characterizes the magnitude of the restoring force that establishes cell activity (De et al 2007) for which the force dipole moment *P* would equal P* (for an infinitely rigid substrate). The first term is the energy the cell invests in deforming the substrate and the second is the cell activity term. Minimizing the effective energy (equivalent to requiring a force balance) yields: 
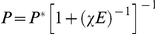
 so that for soft substrates where *χE*<<1, the dipole moment is proportional to the rigidity and can be much smaller than its optimal value, P*, that is favored by the cell activity. Future experimental quantification of the effects of substrate rigidity on the orientational distribution may provide a guide for modifications of the theory that can generalize cell mechanosensitivity to include cell activity, applied stress and substrate deformations in a manner that can predict cell orientation as a function of all these effects.

In line with our observations and the theoretical arguments given here, it has been demonstrated that substrate stiffness influences cell properties, namely cell motility [43], proliferation [44], differentiation [45] and cell organization [46], [47]. Since focal adhesions and stress fibers [48], [49] are affected by substrate stiffness [19], [42], the aforementioned weakening of the cellular contraction on soft substrates appears natural. In summary, the data presented here may explain the fact that cells in very soft environments, as in connective tissue, reorient only negligibly under conditions of stretch compared to those cells embedded in tissues of high stiffness.

Interestingly, at amplitudes a_1_ through a_4_ cells plated on 50 kPa substrates showed distributions of cytoskeletal angles with broad maxima ranging in essence from about 65° all the way to the minimum stress direction (90°) (see Fig. 4). The most likely reason for this is that amplitudes in y-direction are just slightly above 2% upon a_4_ (14%) amplitude in x-direction and therefore below the 3% threshold. To overcome this problem we used the modified ribbon-like chambers without bulky rims on the sides. This allowed us to specifically tune κ to higher values (0.28 instead of 0.15) and thereby to shift the angle of zero strain to smaller angles. At the same time we increased strain values in y-direction to 9.2% (b_5_) from just 4.8% for the box-shaped chambers at identical stretching parameters (a_5_) (see results section and Fig. 4). As result, cells with cytoskeleton orientation close to 90° decreased significantly in b_5_ compared to a_5_ were orientations between 70° and 90° were almost equally present. The amount of reorientation at high angles close to 90° and high strain amplitude can be compared with the results at angles close to 0° and low amplitude. At amplitude a_1_ the strain applied in x direction (0°) is very close to the strain in y-direction (90°) at amplitude a_5_. In both cases we barely observe any reduction in cell number compared to the neutral direction. However, a quite substantial depression is seen both in y direction at amplitude b_5_ (32% strain in x-direction applied to ribbon-like chambers) and in x-direction at amplitude a_2_ (8.4% strain in x-direction). This again corroborates our previous conclusion that cells barely, if at all, react to cyclic strain of amplitudes below 3–4% at the given substrate elasticity of 50 kPa. It also clearly shows that cells orient in the direction of zero strain rather than in the direction of minimal stress.

A detailed analysis of the reorientation process over time revealed differences between cytoskeletal orientation and the overall cell shape. The delayed reorientation of the latter is most probably due to a strict dependence of the cell shape on actin bundle reorientation as primary response. Our observation is in line with the results of other studies [35], [50], [51]. It was demonstrated that cellular shape changes require actin filament remodeling [52] and that among the cytoskeletal components, actin plays a major role in cell shape determination [53]. However, the time lag of several hours between actin fiber and cell shape orientation suggests that the latter is controlled by further mechanisms, e.g. substrate-adhesion remodeling.

As already reported [36], [54], upon axial stretch the cytoskeleton was not only realigned but also reinforced by actin bundling. Because a reinforced cytoskeleton can counteract higher mechanical deformations, this may represent an adaptation of the cells to altered conditions. According to several studies, cells try to maintain a so called tensional homeostasis [40], [55], [56]. This means that cells have an optimal stiffness or force level allowing them to conserve shape. When exposed to environmental changes, they try to minimize these alterations by reorganising their cytoskeleton. However, the literature regarding actin reinforcement in biaxially stretched cells is contradictory [25], [36]. The fact that according to vinculin signals neither the number nor the average size of focal adhesions increased upon stretch indicates that the contact area, i.e. the total area covered by focal adhesions, and thereby most likely also the number of load bearing integrin molecules between the cell and the substrate, was sufficient for cell force transmission. Therefore, the strong increase of vinculin density in focal adhesions might be mainly reasoned by the role of vinculin in focal adhesion to link the increased number of stress fibers [57], [58].

We can currently not explain why actin stress fibers and focal adhesions become reinforced at all although cells tend to reorient in direction of zero strain. Since stress fibers seem to be cross-linked with each other as well as with the surrounding actin meshwork [59] one might speculate that those links undergo strain and therefore might be responsible for enhanced actin bundling. Moreover, due to finite strain effects the angle of zero strain varies slightly by 2.5° at 32% strain. This might result in a small but always present strain applied to stress fibers.

A strong increase of total tyrosine phosphorylation of focal adhesions within the first 30 min of applied stretch followed by a drop to the control level within the following 90 min argues for a pronounced and fast regulation process within the first 2 h of stretching. To our surprise we could not detect a direct succession of tyrosine phosphorylation and actin reorientation, the latter starting to be detectable in our experiments only after 3 hours. These data indicate that high tyrosine phosphorylation levels are not primarily involved in stress fiber or adhesion formation as described before [60]–[62]. The results rather point to very early phosphorylation dependent processes ahead of cell reorientation as e.g. disassembly of existing FAs. Such a process has been already shown to be dependent on enhanced phospho-tyrosine levels [60]. Alternatively, it has been reported that tyrosine phosphorylation of several focal adhesion proteins is indispensible for the induction of stretch induced reorientation by phosphorylation of mechanosensitive proteins [63]–[65].

## Materials and Methods

### Preparation of micro-patterned elastomeric chambers

Elastomeric micro-patterned substrates were prepared by replica molding. Silicon wafers for micro-patterning were produced as described and consisted of a square lattice (lattice constant of 3.5 µm) of small features (Ø 2.5 µm, 300 nm height) [32]. Wafers were fixed in the middle of the chamber molds with the micro-pattern lattice parallel to the chamber sites. Polydimethylsiloxane (PDMS) elastomer was prepared from a two component formulation (Sylgard 184, Dow Corning GmbH, Wiesbaden, Germany). Base and cross-linker were mixed in a ratio of 40 to 1 (by weight) and filled into the chamber mold, forming a 2×2 cm rectangular frame surrounded by a 0.5 cm thick wall and a thin (∼400 µm) micro-patterned bottom (Fig. 1B). These box-shaped chambers with sidewalls were used for all amplitudes marked with an “a” in the text. Alternatively, for several experiments chamber walls parallel to the direction of stretch were omitted during molding. Amplitudes applied to this type of ribbon-like chambers were named with a “b”. After curing over night at 60°C, the material exhibited a Young's modulus of 50 kPa. When needed, a 27–35 µm thin layer of softer PDMS elastomer was prepared on the 50 kPa stiff chamber bottom of box-shaped chambers to obtain substrates with Young's moduli of ∼1 kPa, 3 kPa and 11 kPa. In detail, 2 g of short chain vinyl-terminated PDMS base material (V31, ABCR GmbH, Karlsruhe, Germany) was mixed with 19 µl, 21 µl, and 24 µl, respectively, of cross-linker material (25–35% Methylhydrosiloxane-dimethylsiloxane copolymer; HMS 301, ABCR) and 10 µl of platinum-divinyltetramethyldisiloxane complex (1 to 10 dilution in n-heptan; ABCR) on ice, spin coated onto the chamber bottom at 1600 rpm and cured for 60 min at 60°C. The stiffness of all PDMS elastomers except for ∼1 kPa was calibrated as described [32] using cylindrical test pieces from the same PDMS batches. The elasticity for very soft ∼1 kPa substrates could not be accurately analyzed due to their softness and stickyness. Therefore, the elasticity for those substrates is an estimation based on extrapolating existing calibrations. Layer thickness was determined by confocal microscopy as described in the microscopy section.

Chambers were clamped into the stretching device and uniaxially pre-stretched to avoid sagging of the chamber bottom (Fig.1b). Subsequently chambers were coated with 2.5 µg/cm^2^ fibronectin (Becton Dickinson GmbH, Heidelberg, Germany) in phosphate buffered saline (PBS; 137 mM NaCl, 8 mM Na_2_HPO_4_, 2.7 mM KCl; 1.5 mM KH_2_PO_4_, pH 7.4) for 30 min at 37°C, rinsed with PBS and used immediately for experiments.

### Stretching setup

A linear stage for uniaxial stretch, driven by a direct current motor with integrated gearbox (RB35, Conrad Electronic SE, Hirschau, Germany) was used. Stretch amplitudes were preselected with an accuracy of 20 µm. These amplitudes were stable during the experiment with variations of not more than 10 µm. This provided a triangular stretching function. Additionally, a delay time was programmable for trapezoid stretching functions (Fig. 2, top left).

### Measurement of the principal strains

The detailed description of principal strain measurement is given in the appendix to this paper (Appendix S1). In brief, chambers filled with 500 µl medium were stretched while microscopically analyzed to determine the exact magnitude of deformation in x and y direction of the chamber bottom. The regular lattice of the pre-stretched microstructure was compared with the lattice after an additional defined stretch. The lattice vectors of the microstructure arrays were determined as described in [32] before and after stretching to an accuracy of below 0.02 pixels = 6 nm. This yielded the substrate strain in x- and y-direction accurately. Strain was calculated as relative length change in stretch direction (x) or perpendicular to it (y).

### Cell culture and stretching procedure

Primary human umbilical cord fibroblasts (kindly provided by T. Noll, Bielefeld) were cultured in epithelial cell growth medium (Promocell GmbH, Heidelberg, Germany) supplemented with 100 u/ml penicillin and 0.1 mg/ml streptomycin (Sigma, St. Louis, MO) and kept at 37°C and 5% CO_2_ in a humidified incubator. For all experiments, 10,000 to 15,000 cells of passage 7 to 9 were thawed and seeded on pre-stretched, fibronectin coated elastomer chambers clamped in a stretching device 6 h before stretching. This resulted in a subconfluent layer of isolated cells. The stretching amplitudes amounted to 4.9%, 8.4%, 11.8%, 14% and 32% for box-shaped chambers (in the following named a_1_, a_2_, a_3_, a_4_, and a_5_, respectively) and to 31.7% for the ribbon-like chambers (b_5_) of the pre-stretched chamber bottom length. Stretch and release were applied with a velocity of 10 mm/min with a 3 sec delay between the movements to allow elastomer relaxation. Such constant velocity and therefore strain rate resulted in stretch frequencies between 9 and 52 mHz depending on the amplitude applied. Strain rate as well as delay time were kept identical for all amplitudes to exclude their influence on the results. Stretching experiments were performed at 37°C and 5% CO_2_. Directly after the stretching period, chamber bottoms were glued in the prestretched state onto micro slides. Due to the stickiness of the PDMS elastomer the slides could be adhered to the chamber bottom just by careful pressing against the chamber bottom. Then, cells were subjected to immunofluorescence staining. In parallel, control cells were seeded and kept as described before in a pre-stretched control chamber without further stretch application.

### Immunofluorescence

The immunofluorescence protocol was performed in the PDMS elastomer chamber, with the bottom attached to a micro slide. Cells were fixed in 3.7% formaldehyde (Merck, Darmstadt, Germany) in cytoskeleton buffer (CB; 150 mM NaCl, 5 mM MgCl_2_, 5 mM EGTA, 5 mM glucose, 10 mM 2-(N-morpholino)ethanesulfonic acid, pH 6.1) at 37°C for 20 min. After fixation, cells were incubated with 30 mM glycine (Sigma, St. Louis, MO) in CB for 10 min and washed in CB. Samples were permeabilized with 5% TritonX-100 (Sigma) in CB for 2 min, rinsed in CB and blocked with 5% skim milk powder in CB for 30 min. After rinsing with CB, cells were incubated with 1% primary antibody in CB with 1% skim milk powder for 60 min at 37°C. Unbound antibody was removed by washing the samples three times for 5 min in CB, followed by incubation with 1% secondary antibody in CB with 1% skim milk powder for 60 min at 37°C. Cells were washed in CB, rinsed with water and mounted on coverslips with Gel Mount (Biomeda, Foster City, CA) containing 0.1% (w/v) 1,4-diazabicyclooctane (Sigma). The chamber walls were removed and the samples recorded with fluorescence microscopy performed upside down through the coverslip.

For determination of phospho-tyrosine levels, 20,000 cells per box-shaped chamber were seeded and cyclically stretched with an amplitude of 11.8% for indicated times (see Fig. 7). Directly after, cells were fixed and stained for phospho-tyrosine levels. All these analyses were performed on the same batch of cells. Stainings were done simultaneously for all samples with the same antibody master mix. For microscopic determination of phospho-tyrosine levels, microscope settings were kept identical. From each cell, FAs were marked interactively using ImageJ (Wayne Rasband, U.S. National Institute of Health) and the mean grey values of those adhesion sites were compared.

Primary antibodies used were monoclonal mouse anti-vinculin (clone HVIN-1; Sigma) and monoclonal mouse anti-phospho-tyrosine (clone PY20; BD Biosciences, NJ). As secondary antibody a goat anti mouse antibody coupled to Cy3 was used (F(ab)_2_ fragment; Jackson Immuno Research, Suffolk, UK). Filamentous actin was labeled with 1% phalloidin coupled to Alexa-488 (Invitrogen, Karlsruhe, Germany) simultaneously to secondary antibody incubations in CB with 1% skim milk powder for 60 min at 37°C.

### Microscopy

Thicknesses of PDMS elastomer chamber bottoms were analyzed with a confocal microscope (LSM 510, Zeiss). Z-stacks of pre-stretched chambers were taken using a 50× Epiplan 0.7 NA objective (Zeiss). Imaging was performed in reflection. Due to the different refractive indices of air and PDMS elastomer, the different interfaces (air-PDMS and PDMS-air), gave rise to pronounced reflections which were used to measure the thickness of the elastomer layer. In case of chambers containing two PDMS elastomer layers with different stiffnesses, a fluorescent bead solution (Crimson FluoSpheres 0.2 µm, 1∶2000 in PBS; Invitrogen GmbH, Karlsruhe) was carefully pipetted into the PDMS-chambers before coating with soft PDMS. After 10 min the chambers were washed, dried and the yet uncrosslinked, soft PDMS elastomer was spin coated onto the bead coated bottoms and subsequently cured as described before. Imaging was performed in reflection as well as in fluorescence using appropriate filter sets and a red helium-neon laser for illumination.

Cells were analyzed using an inverted microscope (Axiovert 200, Zeiss) with filter sets appropriate for Alexa 488 and Cy3 visualization with a 40×1.3 NA PlanNeoFluar PH3 oil objective (Zeiss). The micropattern of the chamber bottom was visualized in phase contrast and used to align the chamber lattice parallel to the pixel lines of the CCD camera (ORCA ER, Hamamatsu Photonics, Hamamatsu, Japan). Rows of slightly overlapping images were taken parallel and perpendicular to the direction of stretch along the micropattern, choosing rows along the center of the lattice (Fig. 1B). For all cells within these rows actin and vinculin staining was documented.

To determine the aspect ratios of the stretched cells, completely imaged cells not cut by the limits of the field of view of the camera were prerequisite. Therefore, all samples were also analyzed using an automated cell observer system (Carl Zeiss MicroImaging, Jena, Germany) with the same filter sets and objective as described before. A CCD camera (Axiocam MRM, Carl Zeiss, Jena, Germany) recorded the images. Three to four neighboring rows of adjacent images with 10% overlap were taken automatically. These rows were then stitched using the AxioVision software (Carl Zeiss AxioVision Rel. 4.6.3), resulting in large and highly resolved images of mostly entire cells.

For quantitative comparison of different samples, these were fluorescently stained in parallel and imaged using the confocal microscope with identical microscope settings for all samples. Images were taken using an Antiflex EC Plan-Neofluar 63×1.25 NA oil objective (Carl Zeiss MicroImaging, Jena, Germany) and appropriate filter sets for Alexa 488 and Cy3.

### Image processing

Orientation measurements of actin bundles were done on the first step of the Gaussian pyramid to reduce noise and CPU time. Thus, the original pixel size of 0.1 µm was doubled. The first step of the Gaussian pyramid was calculated by smoothing the original image with a 5×5 binomial mask, sampling every second pixel and smoothing the result with a 3×3 binomial mask. Further preprocessing was performed by high pass filtering with a 7×7 binomial filter, and the high pass result was finally smoothed with a 7×7 binomial mask. The fluorescently labeled cytoskeleton of the cells was segmented by taking all regions brighter than the mean grey value of the image. Only areas larger than 200 µm^2^ (5000 pixels) were considered for further analysis. The orientation of structures in the preprocessed images was then measured with the 2D structure tensor approach [66]. The 2D structure tensor is defined as
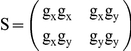
Here, g_x_ and g_y_ represent the x and y-component of the grey value gradient. The angle α between the grey value gradient and the x-axis is given by tan(2α) = 2S_12_/(S_22_−S_11_). To consider only regions with clearly oriented cytoskeleton, the orientation vector, i.e. the unit vector pointing in the same direction as the grey value gradient, was averaged with a 7×7 binomial kernel as weight function. Resulting mean orientation vectors with an absolute value exceeding 0.5 indicated regions of clearly oriented cytoskeleton [67]. As this procedure could not distinguish between different contacting cells in one image, cell edges were marked interactively. Thus, single cells and their cytoskeleton could be identified.

Orientation histograms were created from the cytoskeletal orientations of every single cell. In order to measure the orientation peak, it was firstly ensured that the peak was not located at the edge of the co-domain. This was achieved by splitting the measured histogram at its lowest value and concatenating the parts at the initial interval edges. Secondly, the peak position of cytoskeletal orientations of each cell was determined by smoothing the histograms of all measured orientations with a 59×1 binomial kernel. The position of the maximum value of this smoothed distribution of actin fiber orientations was taken as peak position.

For the determination of cell orientation, the ellipse with the same normalized second central moments as the cell area was determined. The direction of its major axis represented the cell orientation. The aspect ratio was taken as ratio of major and minor axis of the ellipse. This procedure quantifies the overall cell shape whereas the aforementioned analysis of actin fiber directions is based on local structures in immunofluorescence micrographs of the actin cytoskeleton.

The mean grey value of fluorescence intensities of actin, vinculin and phospho-tyrosine staining, respectively, were determined with the software ImageJ (NIH, USA). In case of actin stained cells the grey value of the whole cell was measured by marking the cell body by hand. For vinculin and phospho-tyrosine stained cells the discrete focal structures were also marked by hand.

### Cell force estimation

As detailed in the Results section we observed first cell reorientation at 4.9% substrate strain. Thus, these cells are sufficiently strong to maintain their shape against 4% strain of the substrate. To estimate the forces acting on a cell, we used methods of traction force microscopy [32], [68], [69]. Here, displacements of cell attachments (i.e. focal adhesions) are necessary and were estimated by scaling the x-coordinates with 96%, using the non-moving point of the cell as origin. This is possible because 4% stretching the substrate is equivalent to 4% contraction of a cell on an elastic substrate. Positions of focal adhesions were taken from randomly chosen control data of vinculin labeled cells. Displacements in y-directions were ignored. Our iterative algorithm for force calculation is explained in detail (see Material S1).

### Statistical analyses

Mean grey values of phosphotyrosine staining as well as relative mean grey values of actin and vinculin staining were compared using the student's t-test with a p-value of 0.05.

Actin angle distributions were tested for normal distributions by Kolmogorov-Smirnov and X^2^ –test with a p-value of 0.05. Both tests revealed in all cases non equal distributions. Differences between actin angle distributions were tested for significance using the U-test with a p-value of 0.05.

## Supporting Information

Material S1 **Algorithm for cell force estimation applied to withstand substrate stretch.** Forces were approximated based on the assumption that substrate stretch under an undeformed cell is mechanically equivalent to shrinkage (i.e. contraction) of a cell on an undeformed substrate.
(DOC)

Appendix S1 **Detailed calibration of chamber displacement vector field.** Based on a regular micropattern lattice constants in x- and y-direction allow the exact determination of the transversal shrinkage factor κ (κ = −Δy/Δx) in the plane of the chamber bottom and thus quantify ε_xx_, ε_yy_ and ε_xy_ reliably. Furthermore, the change of angle between the lattice vectors of the micropattern with increasing strain was determined for the box-shaped and ribbon-like chamber.
(DOC)
