# Supplementary material for: Cyclic Stress at mHz Frequencies Aligns Fibroblasts in Direction of Zero Strain
Source: PLoS One. 2011 Dec 16;6(12):e28963. doi: 10.1371/journal.pone.0028963 (PMC3241701; doi:10.1371/journal.pone.0028963)
Supplement: Material S1 — Algorithm for cell force estimation applied to withstand substrate stretch. Forces were approximated based on the assumption that substrate stretch under an undeformed cell is mechanically equivalent to shrinkage (i.e. contraction) of a cell on an undeformed substrate. (DOC) [file pone.0028963.s001.doc]

**Supplementary Material**

**Algorithm for cell force estimation**

Estimation of the force applied to cells during stretching relied on the assumption that cells and their substrates are coupled exclusively at focal adhesion sites. Force estimation was performed using algorithms of traction force microscopy [29, 65]. However, these require as input parameters the coordinates of adhesion sites and the displacements of the elastic chamber material at these sites. Locations of adhesion sites were taken from randomly selected micrographs of vinculin stained control cells. Displacements were calculated based on the fact that 4% stretch of the substrate under an undeformed cell is mechanically equivalent to 4% shrinkage (i.e. contraction) of a cell on an undeformed substrate. For simplicity, deformations in y-direction were ignored. Therewith the deformations in x-direction at the points of force application are determined.

Deformations caused by point forces are not defined at their point of force application due to a mathematical singularity. Therefore three point forces were equidistantly distributed on a circle around artificial focal positions with a radius of 0.2 µm, which is consistent with the radius of small cellular adhesion sites. This way we mimic the deformation at the points of force application. The three point forces were chosen equal in direction and absolute value, and iteratively adapted to reproduce the desired deformation at the center of the circle.

For initialization, three parallel prototype forces f_p_ in x-direction were located on the circle as shown in Fig. S1A. They were arbitrarily chosen as 1 nN each. They caused a prototype deformation u_p_ at the center of the circle, which was used for initialization of the cell force pattern. The non-moving point of the cell was first estimated as the mean position of all focal adhesion sites and used as origin for the focal coordinates. The desired deformations were generated by multiplying the x-coordinates with 96%, which is equivalent to a shrinkage of 4%. The initial three forces for each adhesion site were estimated by scaling f_p_ with the ratio of the deformation at the respective adhesion site and u_p_. However, this initialization neglected the superposition of deformations caused by adjacent forces, resulting in too large deformations due to the initial force distribution (see Fig. S1B). Furthermore, it also yielded a significant sum of all forces caused by a wrong position of the non-moving point. Therefore the resulting deformations were determined with the force pattern used for initialization. The force pattern was iteratively optimized to yield the desired deformations. Additionally the non-moving point of the cell was optimized by a bisection of the x-component of the sum of all forces. This procedure yielded force distributions which produced the desired deformations accurately (relative deviation in x-direction below 2%) and had a resulting force with an absolute value below 2% of the sum of absolute values of all forces.

**
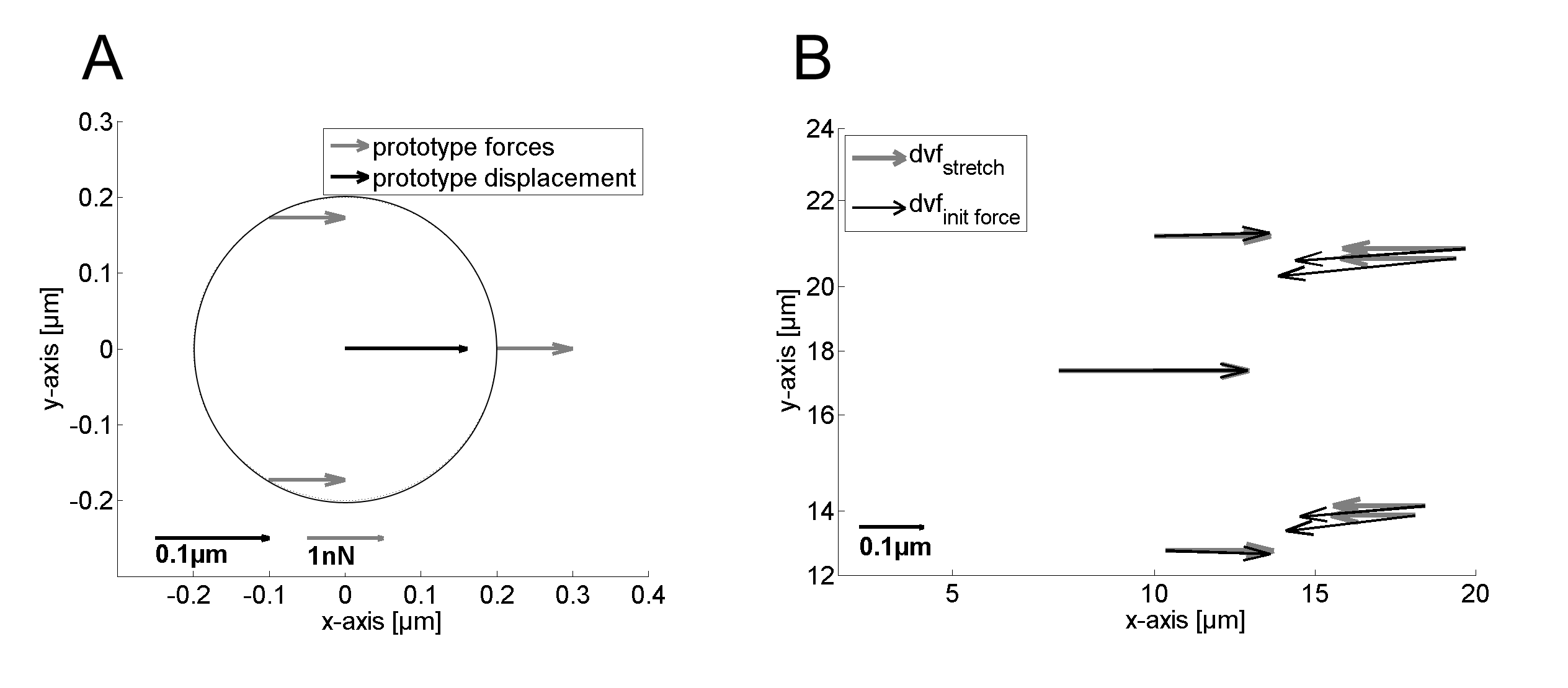
**

**Figure S1 Supplementary Material:** (A) Sketch of three prototype forces f_p_ (gray) of 1 nN parallel to the x-axis, located on a circle of 0.2 µm radius resulting in the prototype displacement u_p_ (black) in the center of about 0.5 µm. The Young’s modulus of the substrate was 50 kPa. The initial force at each focal position was calculated by multiplying the ratio of the desired x-deformation and the x-component of u_p_ with f_p_ regarding the center of all focal positions as non-moving point of the cell. (B) Due to superposition of the deformation fields of the different focal adhesions, the deformations caused by forces calculated as in (A) are higher than those corresponding to a 4% contraction of the cell. To correct this, the non-moving part of the cell and the forces were iteratively optimized until the final displacements differed by less than 2% from the required ones.
