# Supplementary material for: Cyclic Stress at mHz Frequencies Aligns Fibroblasts in Direction of Zero Strain
Source: PLoS One. 2011 Dec 16;6(12):e28963. doi: 10.1371/journal.pone.0028963 (PMC3241701; doi:10.1371/journal.pone.0028963)
Supplement: Appendix S1 — Detailed calibration of chamber displacement vector field. Based on a regular micropattern lattice constants in x- and y-direction allow the exact determination of the transversal shrinkage factor κ (κ = −Δy/Δx) in the plane of the chamber bottom and thus quantify εxx, εyy and εxy reliably. Furthermore, the change of angle between the lattice vectors of the micropattern with increasing strain was determined for the box-shaped and ribbon-like chamber. (DOC) [file pone.0028963.s002.doc]

**Appendix**

**Measurement of the principal strains**

To determine the exact magnitude of deformation in x and y direction of the chamber bottom, i.e. under the cells, chambers filled with 500 µl medium were stretched while microscopically analyzed (Axiovert 200, Carl Zeiss MicroImaging, Jena, Germany) using a 20x 0.3 NA LD Achrostigmat PH1 objective (Zeiss). Phase contrast images were recorded using an ORCA ER CCD camera (Hamamatsu Photonics, Hamamatsu, Japan) and OPEN BOX as software (version 1.77, Informationssysteme Schilling, Munich, Germany). A typical micrograph is shown in Fig. A1.


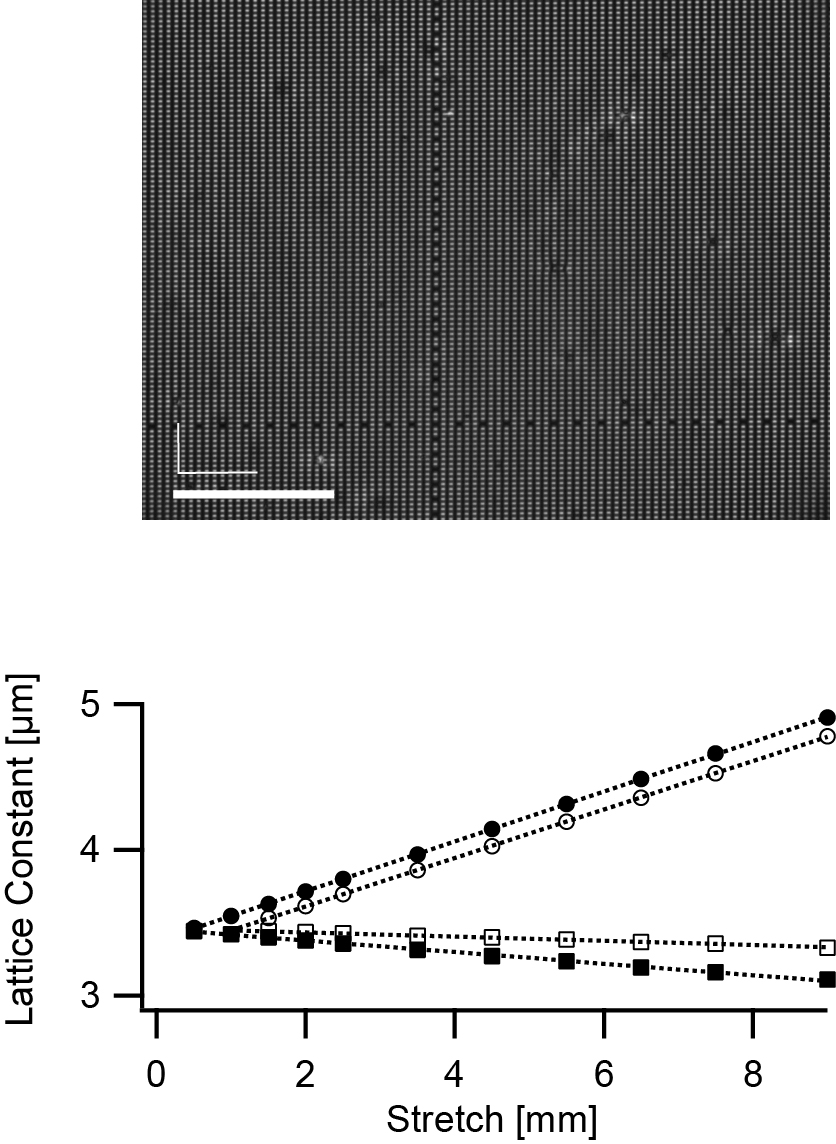


Fig A1: Calibration of cell culture chamber deformation. Top: Micrograph of a strained ribbon-like chamber at 9 mm stretch. The thin white lines point in directions of the lattice vectors of the micromolded grid (for details see text). Their lengths correspond to ten times the respective lattice constant. Here, the lattice constant amounted to 4.91 µm in stretch direction and 3.11 µm in perpendicular direction. Scale bar, 50 µm. Bottom: Lattice constants as function of chamber stretch in direction of stretch (circles) and perpendicular to it (squares). Open symbols indicate box-like chambers, closed ones ribbon-shaped. These data were taken in the center of the chamber.

These micrographs showed the micromolded structure in the chamber bottoms with high contrast. The lattice vectors of these strained grids were determined by cross-correlation of the images with synthetic template functions as described in detail in Cesa et al. [29]. The resulting lattice vectors were highly accurate for two reasons. First, image contrast was high resulting in high precision of the cross-correlation results. Second, in each micrograph several thousand lattice points were located (8697 in the example displayed in Fig. A1). Thus, the final data fit of a regular grid to the lattice points localized by cross-correlation resulted in a final precision of better than 0.02 pixels or 6 nm. During this fit, both lattice constants and the directions of the lattice vectors were determined simultaneously, i.e., the angle between both lattice vectors was not constrained.

Some chambers were calibrated at five positions, one at the center of the microstructure and one at the outermost positions used during experiments on cells, cf. Fig. A2. At all locations and for several values of chamber stretch micrographs were acquired and the lattice parameters were determined. The strain values were calculated using the respective lattice parameters at lowest stretch as reference state. The perpendicular shrinkage ratios, κ, were determined by a linear fit to ε_yy_ as function of ε_xx_. For the ribbon-like chamber displayed in Fig. A2 we obtained values of 0.24, 0.20, 0.20, 0.28 and 0.27 at the center (C), left (L), right (R), top (T) and bottom (B) positions, respectively. For the box-shaped chamber displayed in Fig. A2 the respective values were 0.092, 0.071, 0.076, 0.13 and 0.11. Figure A3 gives an overview over the spatial variation of κ. Taken together the perpendicular shrinkage ratio varied by 0.05 over the analyzed area. The variation between different chambers is of the same magnitude.

Moreover, we analyzed the influence of chamber stretch on the angle between the lattice vectors. The results are presented in Fig. A4. Please note that the shear strain ε_xy_ is simply given by half this angle change (in radians). For a further illustration we fitted the slope of the shear strain ε_xy_ as function of the tensile strain ε_xx_ at each of the measurement locations (cf. Fig A2). The results were quadratically interpolated and are shown in Fig. A5. All these data indicate an essentially shear free deformation. Therefore we neglected shear deformations throughout all calculations.


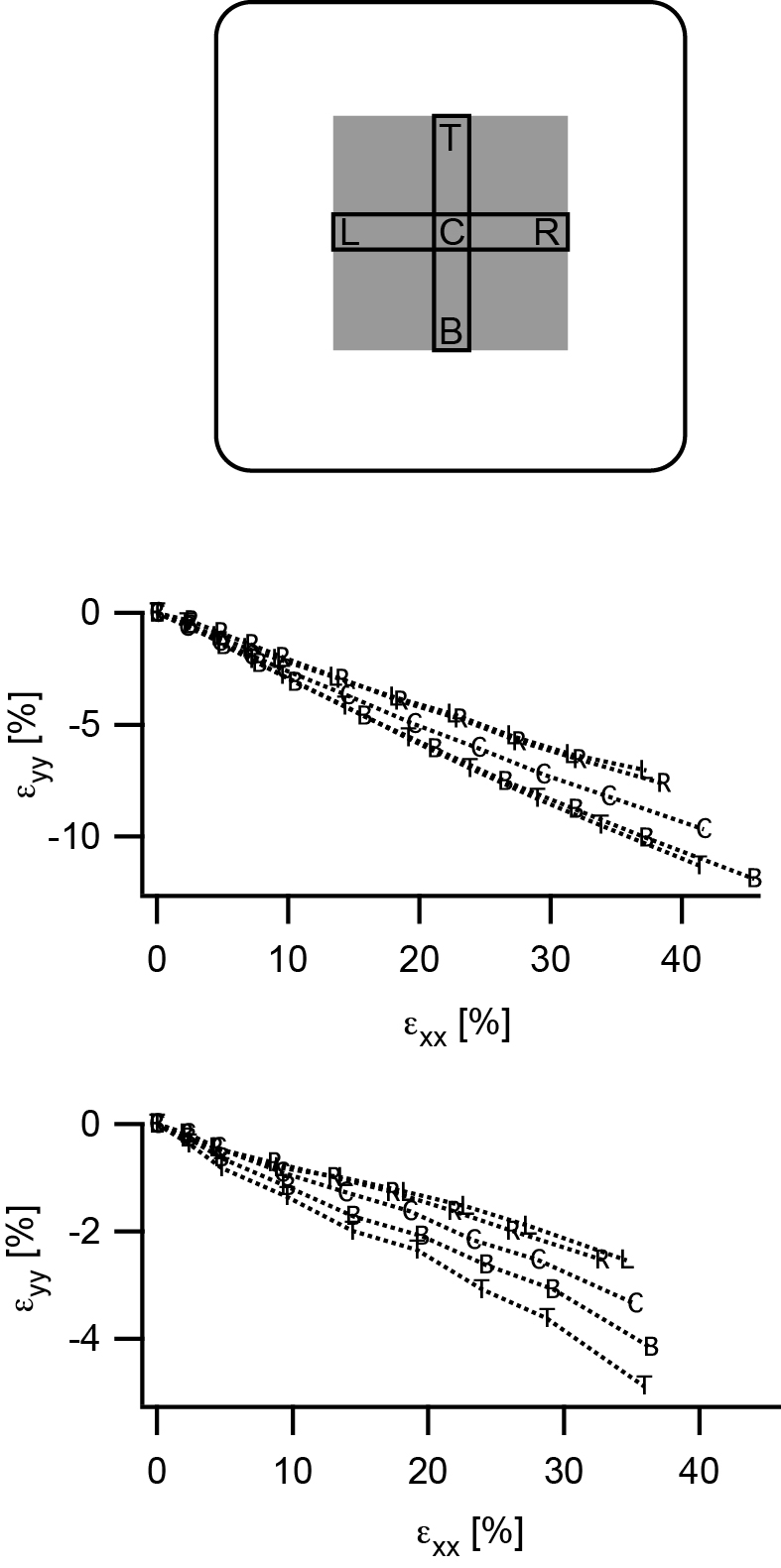


Fig. A2: Chamber calibration. Top: Sketch of the chamber design. Shown are the inner free lamella (a 20 by 20 mm square with rounded edges), the microstructured area (10 by 10 mm square, gray), and the cross-shaped area within which cells were analyzed. The sketch is drawn to scale besides the arms of the cross which are in reality only half as wide. Positions where calibration micrographs were acquired are marked. Middle and bottom: dependence of strain perpendicular to stretch (ε_yy_) on the one in stretch direction (ε_xx_) for a ribbon-shaped chamber (middle) and a box-shaped chamber (bottom). Note the different scales of ε_yy_.


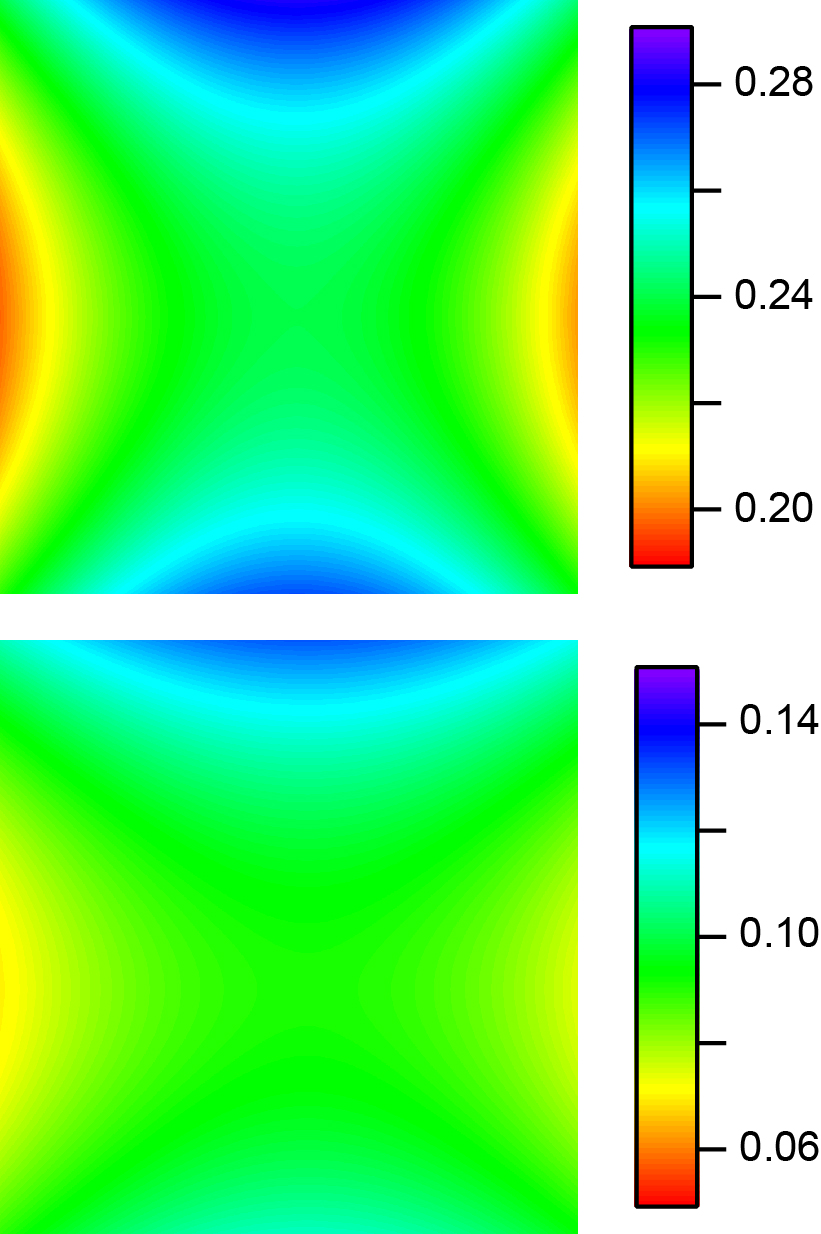


Fig. A3: Spatial variation of the perpendicular shrinkage ratio κ within the microstructured region (10 by 10 mm square) for a ribbon-shaped chamber (top) and a box-shaped chamber (bottom). Here the data shown in Fig. A2 were quadratically interpolated.


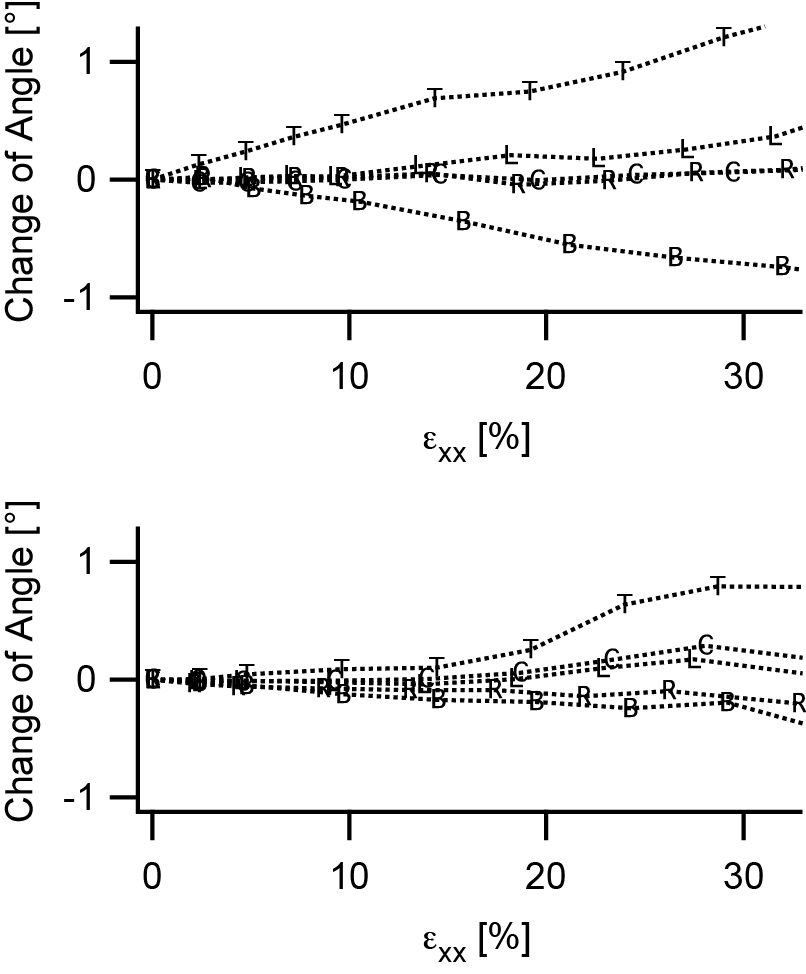


Fig. A4: Chamber calibration. Displayed is the change of the angle enclosed by the lattice vectors during straining. Top: ribbon-like chamber, bottom: box-shaped chamber. Characters indicate the location within the chamber as indicated in Figure A2.


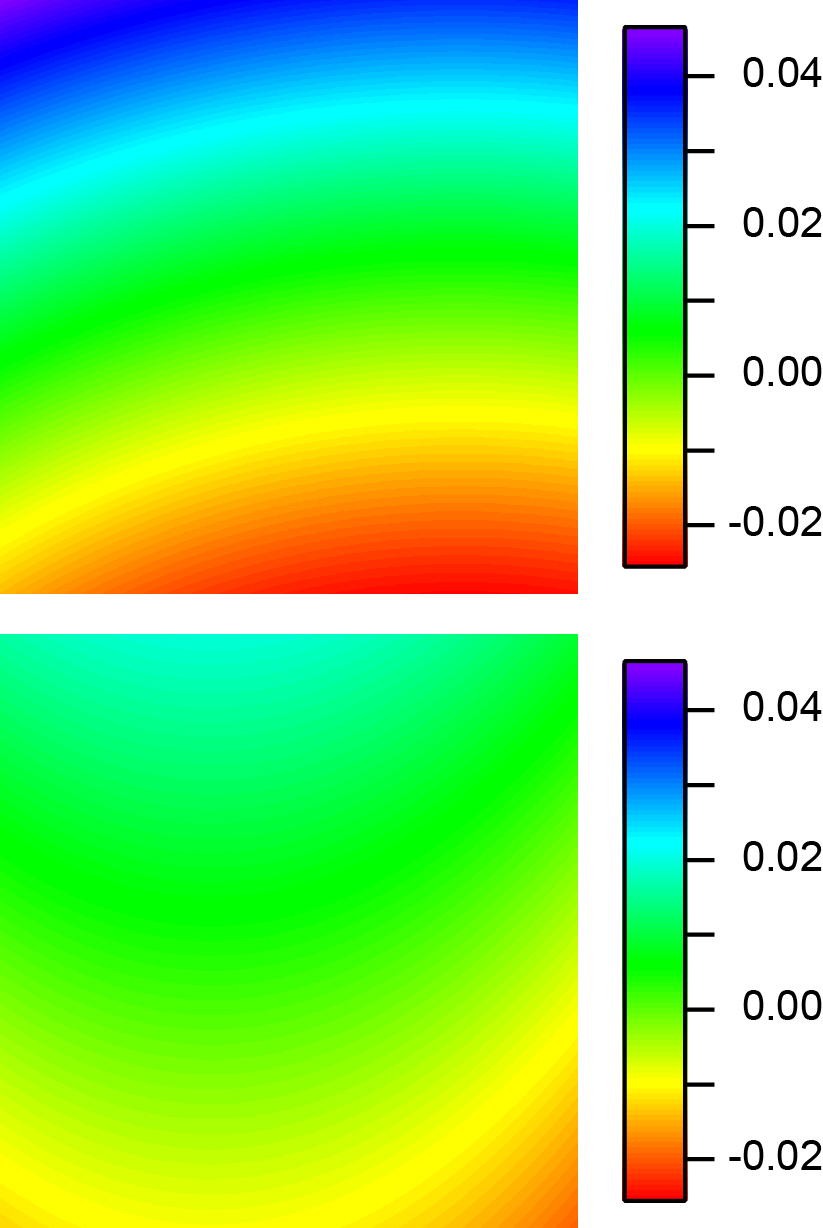


Fig. A5: Spatial variation of ε_xy_/ε_xx_ over the microstructured area (10 by 10 mm square) for a ribbon-shaped chamber (top) and a box-shaped one (bottom). These images were calculated from the data presented in Fig. A4, for details see text.
